# Bioimaging guided pharmaceutical evaluations of nanomedicines for clinical translations

**DOI:** 10.1186/s12951-022-01451-4

**Published:** 2022-05-19

**Authors:** Ruslan G. Tuguntaev, Abid Hussain, Chenxing Fu, Haoting Chen, Ying Tao, Yan Huang, Lu Liu, Xing-Jie Liang, Weisheng Guo

**Affiliations:** 1 Department of Minimally Invasive Interventional Radiology, Key Laboratory of Molecular Target & Clinical Pharmacology, School of Pharmaceutical Sciences & the Second Affiliated Hospital, Guangzhou Medical University, Guangzhou, 510260 People’s Republic of China; 2 Advanced Research Institute of Multidisciplinary Science, School of Life Science, School of Medical Technology (Institute of Engineering Medicine), Key Laboratory of Molecular Medicine and Biotherapy, Key Laboratory of Medical Molecular Science and Pharmaceutics Engineering, Beijing Institute of Technology, Beijing, 100081 China; 3 Department of Hepatobiliary and Pancreatic Surgery, Affiliated Hospital of Nantong University, Nantong, 226001 China; 4 Chinese Academy of Sciences (CAS) Key Laboratory for Biomedical Effects of Nanomaterials and Nanosafety, CAS Center for Excellence in Nanoscience, National Center for Nanoscience and Technology of China, Beijing, 100190 People’s Republic of China

**Keywords:** Nanomedicine, Biomedical imaging, Pharmaceutical evaluations, Physicochemical characteristics

## Abstract

Nanomedicines (NMs) have emerged as an efficient approach for developing novel treatment strategies against a variety of diseases. Over the past few decades, NM formulations have received great attention, and a large number of studies have been performed in this field. Despite this, only about 60 nano-formulations have received industrial acceptance and are currently available for clinical use. Their in vivo pharmaceutical behavior is considered one of the main challenges and hurdles for the effective clinical translation of NMs, because it is difficult to monitor the pharmaceutic fate of NMs in the biological environment using conventional pharmaceutical evaluations. In this context, non-invasive imaging modalities offer attractive solutions, providing the direct monitoring and quantification of the pharmacokinetic and pharmacodynamic behavior of labeled NMs in a real-time manner. Imaging evaluations have great potential for revealing the relationship between the physicochemical properties of NMs and their pharmaceutical profiles in living subjects. In this review, we introduced imaging techniques that can be used for in vivo NM evaluations. We also provided an overview of various studies on the influence of key parameters on the in vivo pharmaceutical behavior of NMs that had been visualized in a non-invasive and real-time manner.

**Graphical Abstract:**

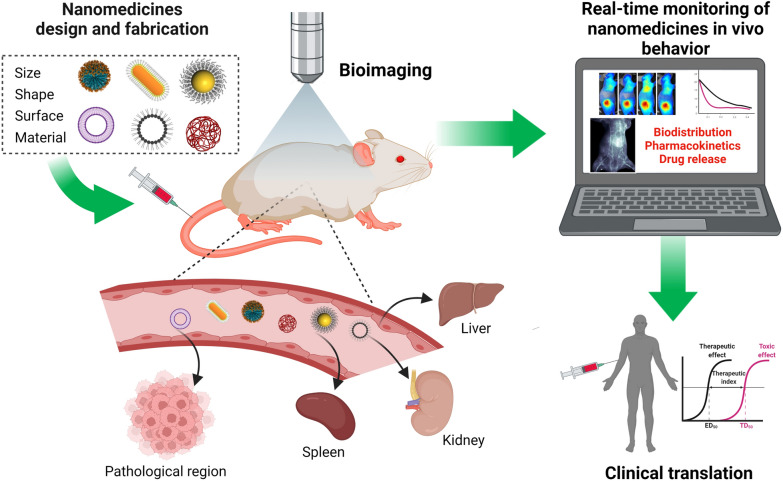

## Introduction

In alignment with rapid advances in biomedical and pharmaceutical sciences in the last few decades, the unprecedented development of nanotechnological approaches has greatly promoted the emergence of nanomedicines (NMs) exhibiting high diagnostic/therapeutic efficacy and improved safety profiles [1]. NMs are intended to maximize therapeutic/diagnostic efficacy while avoiding off-target and accumulation-related adverse effects by delivering therapeutic or imaging agents (passively or actively) to the tissue of interest [2]. Therapeutic agents in the nanoscale size range have found wide applications in the treatment of ailments such as cancer, chronic inflammation, or vascular diseases [3–5]. Due to the small size and high surface-area-to-volume ratio, the NMs exhibit remarkable differences in chemical reactivity, fluorescence, magnetic permeability, and electrical conductivity compared with conventional bulk chemical equivalents [6]. These unique properties can greatly enhance the range of possibilities in the development of innovative drugs. However, these properties can also bring about additional challenges and limitations related to the quality, safety, and efficacy of the nanoscaled products. The physicochemical properties of the NMs can alter pharmacokinetic profiles, changing their absorption, distribution, metabolism, and elimination, which results in general concerns on the application of NMs [7]. The interaction of NMs with their biological surroundings (at molecular, cellular, and organ levels) greatly depends on the complex interplay of the controllable properties of the nanoparticles and the largely uncontrollable properties of their surroundings. Particle size, shape, and surface chemistry are key factors governing the performance criteria, including the degree of protein adsorption, cellular uptake, biodistribution patterns, and clearance mechanisms [8]. NMs have the potential to be precisely designed by tuning these factors to achieve individualized and more efficient treatment and diagnostic agents while minimizing potential side effects [9, 10]. As the NMs landscape evolves, it is increasingly important to understand the intrinsic connection between the structural and functional relationship, which allows the further optimization and manipulation of fine nanostructures to meet general standards, applicable to medicinal compounds.

Along with traditional and biological drugs, the approval process of NMs is regulated by the Food and Drug Administration (FDA) in the USA. Therefore, NMs are usually subjected to the standard range of preclinical and clinical validation. Following the discovery of the material, the preclinical phase of testing generally involves animal studies that investigate efficacy, safety, and toxicity profiles, as well as appropriate dose ranges [11]. When dealing with NMs, it is of great importance to achieve a comprehensive understanding of their physicochemical characteristics, and significant research is still required to evaluate their behavior in biological systems, including the use of both new assays and existing methods. Despite numerous research activities in academia and clinics, there are very limited NMs approved for clinical usage, whereas more than 400 nano-engineered products are currently in ongoing clinical trials. The pharmaceutical evaluation of NMs remains a key issue in several guidance documents published by the FDA over the past few years, which is also an important aspect of research and development [12]. Thus, the translation of novel concepts like NMs into commercially viable products for clinical application requires rigorous evaluation on the basis of regulatory guidance regarding their quality, safety, and efficacy.

**Fig. 1:**
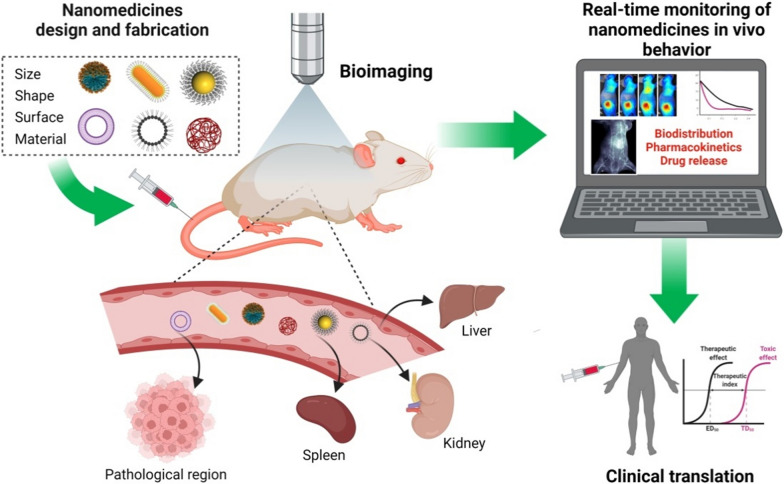
Non-invasive imaging modalities can provide real-time monitoring of NMs fate in vivo and facilitate their clinical translation. Created with BioRender

Due to the physicochemical complexity of NMs related to their size, shape distribution, surface chemistry, single/multiphasic composition, and presence of cognitive groups, it remains a formidable challenge to investigate their pharmaceutical profiles in a biological environment using conventional pharmaceutical evaluations. To address this challenge, various efforts have been made to perform *in situ* visualization of metabolic behaviors of systemically administrated NMs with newly-developed imaging modalities and technologies [13–17]. The ability to account for the total dose administered and its fate is of great importance in providing efficiency and safety. Current methods of in vivo imaging mainly include introducing chemical tags – fluorescent probes, isotope labels, and photoacoustic (PA) agents, amongst others – into NMs for tracing. Unlike conventional methods used for pharmaceutical evaluations, the non-invasive nature of biomedical imaging modalities (e.g., magnetic resonance imaging, positron emission tomography, computed tomography) allows for direct monitoring and quantification of the pharmacokinetic and pharmacodynamic behavior of labeled NMs in a real-time manner. The use of in vivo imaging methods in pharmaceutical evaluation can significantly improve the efficiency of NMs and the commercialization of innovations, taking them from laboratory concept to clinical practice (Fig. 1). In this review, we have discussed the influence of key parameters of NMs on their in vivo behavior that had been monitored in a non-invasive and real-time manner.

## Clinical translation status of NMs

**Fig. 2:**
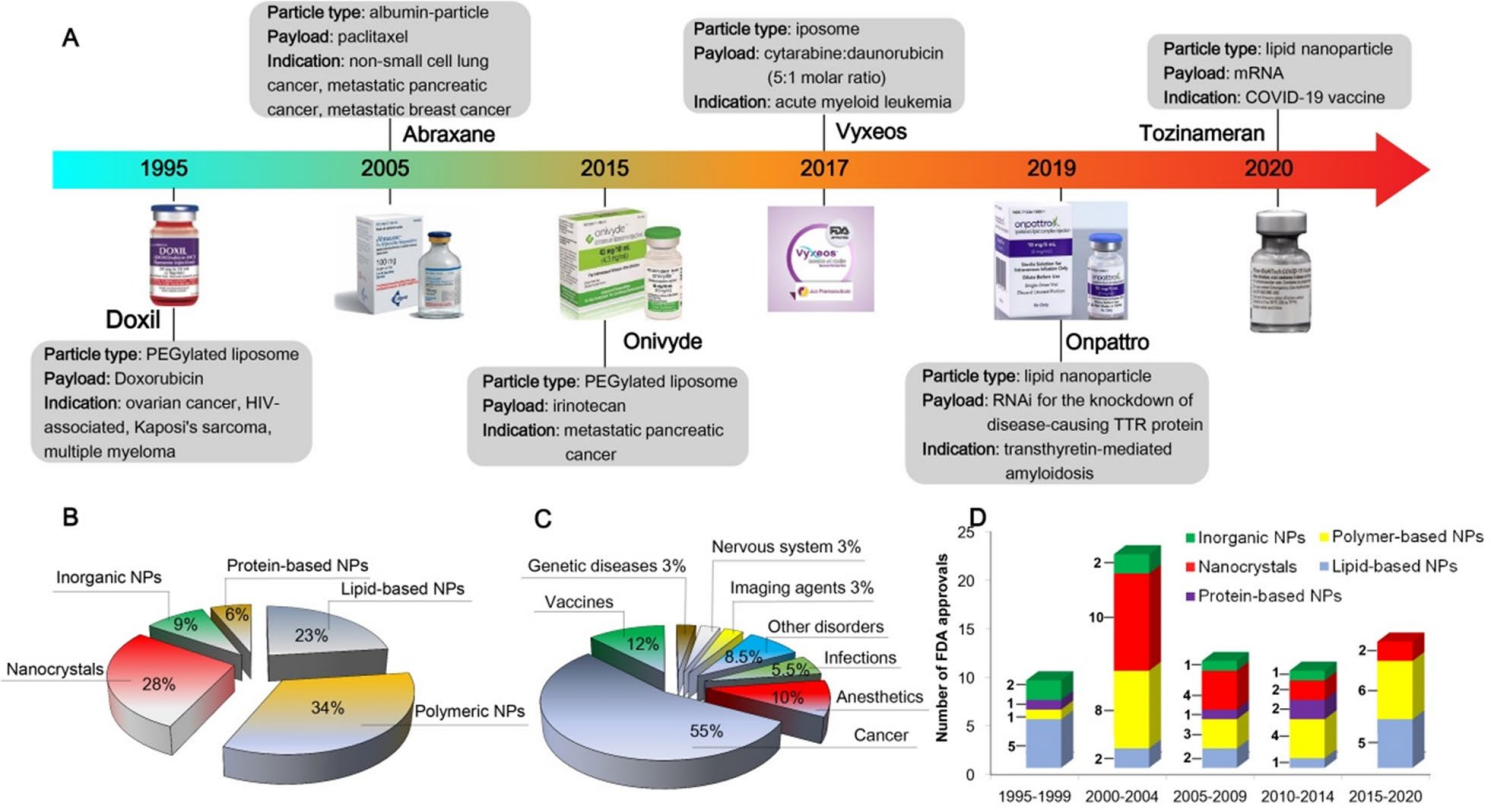
Clinical translation status of NMs. **A** Timeline of the development of major NMs. **B** clinically approved NMs categorized by particle type. **C** categorization of current clinical trials of NMs based on indications. **D** сhronological NMs approvals categorized by particle type

The use of NMs has great potential to make significant impacts on human health through the prevention, diagnosis, and treatment of diseases. NMs have shown various advantages, such as crossing or penetrating biological barriers and delivering hydrophobic and biological drugs with high efficiency at the target site. The first NM approved by FDA for clinic application was named Doxil^®^, which exhibited prolonged drug half-life and minimized uptake by the mononuclear phagocyte system (MPS) due to the use of PEGylated liposomes [18]. Afterward, more than 60 NMs have received FDA approval for clinical applications (Fig. 2A). These marketed NMs are used for treatments of various diseases, including cancer, fungal disease, iron-replacement therapies, macular degeneration, anesthetics, and rare genetic diseases [19–21]. The majority of clinically approved classes of NMs involve nanocrystals, lipid-based, polymer-based, protein-based, and inorganic nanoparticles (NPs) (Fig. 2B). Given the large number of clinical trials of NMs in progress – over 400 NMs and increasing approved new generations of NMs will enter the market in the near future [22]. Although the ongoing clinical trials are mainly focused on cancer treatment, other clinical uses of NMs related to infectious diseases, pain treatment, vaccination, and imaging are also being tested for clinical introduction (Fig. 2C). Furthermore, new areas for treatment are emerging and encompass disorders associated with neural system diseases, eye diseases, and genetic diseases [22].

Despite the enormous amount of research reports, the commercially available NMs are still very limited. There exists a major gap between laboratory research and the clinical practice of NMs due to various challenges that arise throughout the whole stages of development. Several shortcomings have been identified as key scientific issues related to delivering NMs to patients, including the analysis and characterization of NMs, biological challenges, large-scale manufacturing, biocompatibility, and safety [23]. One of the major concerns for the translation of NMs to the clinic is the deficient understanding of NMs’ in vivo behavior. Compared with conventional pharmaceutical formulations, NMs have more complicated physicochemical features, including size distribution, morphology, surface properties, chemical composition, and stability. Thus, it is highly challenging to analyze the relationship between their physicochemical properties and their behavior in biological systems. For instance, even if NMs with similar average-sized particles have different polydispersities, it may cause dramatic alternations to in vivo behaviors such as biodistribution, targeting ability, drug release rate, and toxicity [24, 25]. Typically, the development of a nanoengineered product is based on a fabrication-driven approach, where a novel NM is first designed and characterized from a physicochemical perspective. Limitations in the clinical translation are revealed only when attempting to align the newly developed medicines with their biological applications. The key determinants of the successful clinical translation of NMs are their distribution, accumulation in the target tissues, retention, and efficacy as well as the correlation between NMs’ physicochemical characteristics and in vivo fate in an animal model compared to humans. From the design of NMs, it is essential to consider the physicochemical features of various NMs in overcoming biological barriers in order to ensure highly efficient targeting of the region of interest and reduced accumulation in unwanted sites [26]. Evaluations of in vivo behavior of NMs in different preclinical animal models, which represent aspects of specific diseases, are preferred for gaining a comprehensive understanding of the intrinsic structure-function correlation.

## Biomedical imaging used for in vivo pharmaceutical evaluations of NMs

The characterization of NMs requires a combination of various techniques to understand their physicochemical features and behavior in biological systems [19, 27]. From a regulatory perspective, conventional medicines are analyzed with well-established assay systems, while NMs should follow their own framework that accounts for their complexity to establish new guidelines defining properties specific to nanomaterials. Since most of the challenges associated with the pharmaceutical evaluation of NMs arise from their structural and physicochemical complexity (e.g., size, shape, coatings, payload release) traditional approaches for drug regulation cannot be applied without a substantial adaptation to the nature of nanotechnology-related products. Pharmacological and toxicological profiling require a systematic assessment of the NMs’ behavior in different kinds of in vivo systems depending on their physicochemical properties. Therefore, the combination of physicochemical characteristics and physiological conditions determines the biological application of NMs.

**Fig. 3:**
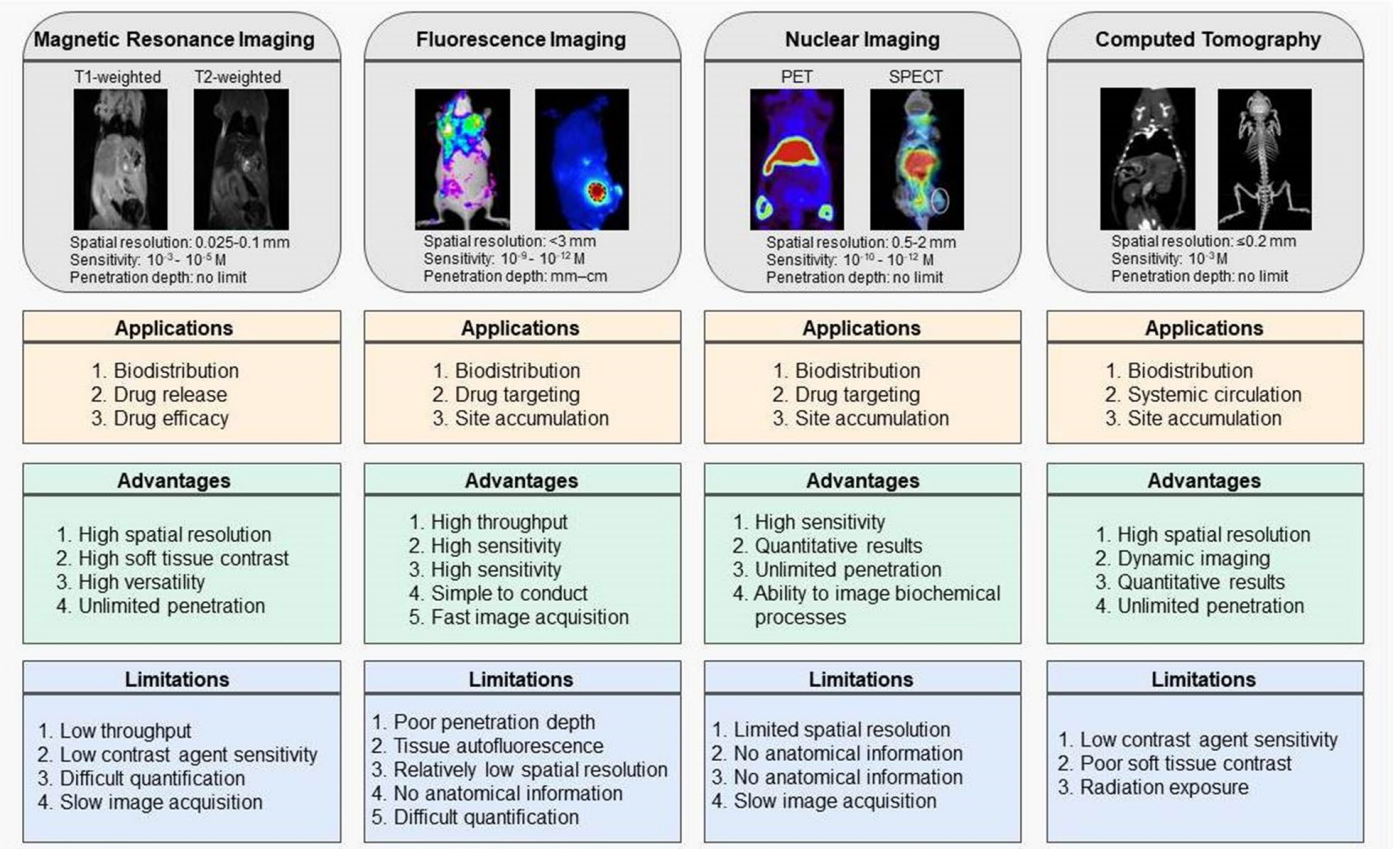
An overview of specific applications, advantages, and limitations of imaging modalities applied in NMs research. Adapted with permission from [28–34]. Copyright 2014, 2015, 2019, 2021, American Chemical Society; 2019, 2020, Springer Nature; 2015, Elsevier

To provide fundamental insights and to ensure more accurate delivery of drugs to various pathological sites, it is crucial to monitor multiple aspects of the drug delivery process quantitatively, including its biodistribution, pharmacokinetics, target site accumulation, localization in the target site, distribution to healthy organs, drug release kinetics, and therapeutic efficacy. In this context, there is growing interest in the use of non-invasive imaging modalities – e.g., magnetic resonance imaging (MRI), fluorescence imaging (FLI), positron emission tomography (PET), single photon emission computed tomography (SPECT), and computed tomography (CT)–for the real-time monitoring of NMs’ biodistribution, pharmacokinetics, drug release, and therapeutic efficacy [28]. An overview of these imaging modalities is presented in Fig. 3. The increasing use of these techniques for pharmaceutical assessment has led to the development of materials that serve as contrast agents for imaging modalities. Below, we have briefly described commonly used imaging modalities and materials serving as imaging probes that can be used for in vivo visualization and that possess a great potential for pharmaceutical evaluations.

### Magnetic resonance imaging

Magnetic resonance imaging (MRI) is a non-invasive imaging technique that provides detailed physiological and anatomical information within living tissues. It is based on the same principle as chemical nuclear magnetic resonance (NMR), detecting the spinning and rotation of particular atomic nuclei inside the organism. Generally, the MRI signal is derived from endogenous mobile water protons, which are presented in the subject’s body. When the strong static magnetic field is applied to a subject, the magnetic moment associated with protons tends to align with the direction of the magnetic field, and the nuclei of the protons are brought out of this equilibrium by pulsed radio-frequency (RF) radiation. After the RF radiation is removed, the nuclei are returned to equilibrium and induce a transient voltage, generating the NMR signal in the receiver antenna. The alignment of the nuclei along the direction of the magnetic field does not occur instantly, but gradually over a period of time parameterized by spin–lattice relaxation time (T1). Spin–spin relaxation time (T2) is the time constant that characterizes the period of time during which nuclei remain in ‘phase’ with each other. The amount of available signal is intensely dependent on the physical characteristics of a specific region, such as nuclei density [35]. MRI is able to provide more specific details that other imaging methods are not able to access because its penetration depth is adequate to view the entire body, with a spatial resolution of up to 10 μm [36]. Despite its widespread use for the diagnosis of disease and monitoring of therapy, MRI is also commonly applied in the research of NMs to analyze biodistribution and pharmacokinetic profiles [37]. The standard MRI, however, has low sensitivity since it is often used for anatomical reasons without contrast. The low sensitivity is related to the typical signal detection mechanism of the relaxation rates of protons contained in the water and fat within tissues [38]. If one desires to enhance the detection levels and obtain molecular information, contrast agents (including nanomaterials) can be used in micromolar and even in nanomolar concentrations to modify the water proton relaxation rates [39, 40].

#### MRI imaging probes

MRI contrast agents are typically divided into two groups based on the physical mechanism. This process enables the MRI contrast agents to generate signal: T1 (longitudinal relaxation time) and T2 (transverse relaxation time). T1 contrast agents are typically paramagnetic species that shorten longitudinal relaxation time leading to brightening on T1-weighted MR images on T1-weighted MR images and represent the ‘positive’ contrasts. These contrasts emerge from the magnetically non-linear spin layers that present mainly on the surfaces of magnetic objects. At these surfaces, many unpaired electrons are retained by metal ions that help accelerate T1 relaxation [41]. One of the most commonly exploited T1 contrast agent is Gadolinium (Gd^3+^). Gd^3+^ - chelated complexes have been used for diagnostics for the last 30 years and have continued to be studied for improved and more functional applications. The cause behind Gd^3+^ as a great T1 agent is due to its seven unpaired electrons that effectively reduce T1 values resulting in sufficiently lengthy electronic relaxation times, as a consequence generating signal that is more long-lasting [42–44]. While most of Gd^3+^-based imaging agents are chelates with small molecules that may display heavy metal-associated toxic effects due to leaching processes, nanomaterials that incorporate Gd^3+^ exhibit substantially less leaching, resulting in improved safety profile [45]. For instance, such nanomaterials as gadolinium phosphates (GdPO_4_), gadolinium oxides (Gd_2_O_3_), and GdF_3_:CeFn_3_ have been fabricated to achieve improved in vivo behavior in MRI [46–49]. In order to avoid renal toxicity in kidney failure patients and other adverse effects of Gd^3+^, other T1 contrasting materials, such as Manganese (Mn^2+^) has been studied. Mn^2+^- based imaging agents are considered to have ideal characteristics for MRI because of their ability to overcome toxicity issues, short circulation half-life, and low intracellular accumulation that other T1 contrast agents suffer [50]. Manganese oxide (MnO) and other materials displayed negligible toxicity and good T1 weighted contrast effects and has been used as preclinical in vivo T1 agents [51–53]. However, it was revealed that the geometry and morphology of MnO nanomaterials critically affects their relaxivity, since the interaction with water strongly influences contrast effects [54]. The fact that T1 imaging agents generate contrast depending on approach to freely diffusing surrounding water molecules, provides suitable conditions for assessing drug release as they render distinctive signals when loaded or released from the drug delivery system [55].

T2 imaging agents are typically materials that reduce the contrast of the images under consideration, to provide ‘negative’ contrast. Majority of the magnetic nanomaterials are present by iron-based compounds that exhibit a high magnetization, which can cause magnetic inhomogeneities affecting T2 relaxation [56]. In particular, the mechanism of MR T2 contrast is based on the local magnetic field gradients that are produced by iron oxide materials for their dipoles, which are generated by spins of some of their electrons in order to interact with water protons. After the radiofrequency pulse is ceased, the dipolar connection amongst the water proton spins and the iron oxide magnetization accelerates the pace about which protons are pushed out of phase with each other, lowering T2 relaxation periods. Shorter T2 relaxation reduces signal intensity resulting in darkening and negative image contrast [57]. Iron oxide materials mainly comprise hematite (α-Fe_2_O_3_), magnetite (γ-Fe_2_O_3_), and magnetite (Fe_3_O_4_). All types demonstrate size-dependent properties and can be roughly divided into three groups: monocrystalline iron oxide NPs (MIONs), superparamagnetic iron oxides (SPIOs), and ultrasmall superparamagnetic iron oxides (USPIOs). These relate to varied size ranges, however the composition and structure also play a vital role. Iron oxide NPs were used in the wide variety of investigations for cancer and atherosclerosis detection, lymphoid tissue imaging, and tracking cells such as immune cells and stem cells throughout the body [58–62]. Aside from pure iron oxide, a number of iron alloys such as FeCo, CoFe_2_O_4_, MnFe_2_O_4_, NiFe_2_O_4_, FePt, and others, have been fabricated to enhance T2 signal and produce high contrast [63–67]. These contributed to improved visualization of very small structures, which helped in more accurate diagnose and assess diseases. Throughout history, iron oxide has been used in human beings, considered safe, and a large number of techniques have been developed to fabricate particles with varying range of sizes, structures, and combinations with other materials. Recent modification of fabrication chemistries achieved specifically concise control of standard iron oxides size, crystallinity, surface properties, and uniformity resulting in substantially improved in vivo behavior and imaging properties [29, 39, 68, 69]. Although iron oxide is extensively used for iron-based imaging agents, current achievements have greatly expanded the iron state space and all magnetic materials. These newly engineered contrast agents possess higher saturation magnetizations and magnetic moments resulting in improved preclinical magnetic imaging by enhancing image contrast and reducing the amounts obligatory for administration.

### Fluorescence imaging

Fluorescence imaging (FLI) has become an essential tool for evaluating the biodistribution and target site accumulation of NMs due to its ease of use and excellent contrast agent sensitivity [70, 71]. FLI is based on photon-electron interactions and their resulting electron energy states. It exploits differential electron states stimulated by incoming excitation light, leading to emission of a longer-wavelength photon. Generally, the fluorescent process involves the absorption of light by tissues, followed by the emission of some of this light, which is captured with a detector that converts this information into images. The aim is to separate the emitted light from the excitation light [72]. The key to the visualization of living objects with FLI is maximizing penetration depth and minimizing background signal while maintaining signal resolution and intensity. This can be achieved by using different approaches, which includes changing the wavelength of light and enhancing intensity and contrast by multiple near-infrared (NIR) fluorophores [73, 74]. Compared with visible light, NIR fluorescence provides less tissue scattering and absorption, resulting in much higher penetration efficiency and reduced non-specific background autofluorescence interference from biological tissues [75–78]. NIR-based imaging approaches offer relatively accurate information on the localization of fluorophore-labeled NMs and allow for quantitative biodistribution assessment. The sensitivity, versatility, and ease of FLI, along with its quality of displaying several imaging probes in the same animal, are the most significant advantages of this modality. Despite the wide use of non-invasive FLI techniques in preclinical studies, some formidable hurdles still limit its popularity in clinic and industry pharmaceutic development, including its autofluorescence and insufficient penetration depth [28]. Optical agents are essential for light absorption in photo-induced imaging because, in an aggregation state, they often display redshift absorption and emission in NIR, which is advantageous for deep tissue disorders that require more light penetration.

#### FLI imaging probes

The variety of fluorescent substances used in the standard NIR window (NIR-I: 650–900 nm) has been widely utilized in both animal and clinical applications since the FDA approved the first NIR dye, indocyanine green (ICG) [79–81]. Cyanine fluorophores possess high absorption coefficients, and their chemical groups can be modified for further improvement of optical properties [82]. Several cyanine dyes in the NIR-I region have been approved in recent years for commercial use, including DiR, Cy7.5, Cy7, IR-125, IR-140, IR-775, IR-780, IR-783, IR-797, IR-806, IR-820, IR-830, IR12-N3, IRDye800cw, HITCI, etc. However, tissue autofluorescence, quality of photon attenuation, and scattering are all relatively high when imaging at shorter wavelengths, hindering further development of NIR-I agents.

Recently, the second near-infrared (NIR-II: 900–1700 nm) fluorescence imaging method demonstrated increased imaging depths and temporal-spatial resolutions, with a greater ratio of signal and noise compared to the NIR-I window [83–88]. These significant improvements have given a new impetus to investigate novel NIR-II materials, further exploring their potential for biomedical applications including metabolism monitoring and NMs distribution in deep body tissues. Semiconducting single-walled carbon nanotubes (SWNTs), which have been employed for targeted probing of cell surface receptors and demonstrated great accuracy and specificity, were produced as one of the first NIR-II fluorescent tags with narrow band gaps [89]. After that, a wide range of optical nanomaterials that possess small effective masses and narrow band gaps, such as semiconductor quantum dots (QDs), rare-earth doped NPs, and other inorganic nanomaterials (e.g., gold NPs, mesoporous silica) had been developed [90–98]. However, inorganic nanomaterials suffer from safety issues regarding immune uptake and clearance after imaging [99]. Organic dyes, on the contrary, represent an exceptional alternative, as they offer significant biocompatibility and biosafety advantages, and their fluorescence properties can be controlled by rational chemical structure design. For example, semiconducting polymers and small molecules with an alternating electron deficient group (acceptor, A) and electron abundant group (donor, D) exhibit sharp and strong absorption and emission in the NIR-II window [100–102]. The electron acceptor with electron donor groups through π-bridging moieties. This spatial configuration of strong electron donor groups adjacent to the central electron acceptor serves to reduce the energy gap separating the hybridized levels of the highest occupied molecular orbital (HOMO)/lowest unoccupied molecular orbital (LUMO) and shifts the fluorescent emission to the NIR-II region. Reducing the band gap between the HOMO and the LUMO is an effective method to obtain D–A semiconducting molecules [103, 104]. Acceptor groups are usually presented by benzobisthiadiazole (BBTD) that has a significant quinoidal character, which allows to improve the delocalization of electrons. For the donor groups, fluorine, thiophene, and their derivatives have been developed to form the D–A–D fluorophores. To make these structures water-soluble, they are either loaded into hydrophilic polymer matrices or conjugated with polyethylene glycol (PEG) [105–109].

### Nuclear imaging

Positron emission tomography (PET) and single-photon emission computed tomography (SPECT) are currently the most used nuclear imaging procedures. Both modalities require the administration of radioactive tracers. PET is an imaging modality in which radionuclide emits positrons that annihilate nearby electrons, thereby generating two γ photons with an energy of 511 keV that are identified by detectors embedded in PET scanners. In contrast to PET, the radiotracer used in SPECT emits γ-rays that are measured directly and the energies used in SPECT are varied, and energy-dependent imaging allows simultaneous evaluation of different radioactive tracers and therefore different radioactively labeled nanomaterials [110]. Nuclear-based visualization techniques, like those of magnetic materials, show remarkable advantages due to their ultrahigh sensitivity and unlimited penetration through the whole body and have a great potential to be used at preclinical level for monitoring biodistribution and pharmacokinetics of administered NMs. The ultrahigh sensitivity of radio isotopic nuclear imaging is a key benefit; only “trace” quantities of radioisotope are required to produce a sufficient signal level. The selection of radiolabel is influenced by the physical properties of the isotope, like positron energy (which determine its travelling distances in tissue, and thus the spatial resolution of the image formed), decaying half-life, and the effectiveness of the radiolabeling method. Theoretically, any type of NMs can be tagged with radionuclides, pinpointing the exact location of their accumulation. Three approaches – surface coupling, interior integration, and interface engineering–have been widely employed to label NMs with radionuclides [111].

#### Nuclear imaging probes

Surface coupling is based on the decoration of nanomaterials surface by radionuclides. Typically, two strategies are applied for carrying out this process, namely the indirect surface labeling and the direct surface labeling. In the first strategy, certain chelators such as diethylenetriaminepentaacetic acid (DTPA), 1,4,7,10-tetraazacyclododecane-1,4,7,10-tetraacetic acid (DOTA), 1,4,7-triazacyclononane-1,4,7-triacetic acid (NOTA), and deferoxamine (DFO) are anchored on the top layer of NMs for linking with metallic radionuclides (e.g., ^99^mTc, ^177^Lu, ^64^Cu, ^68^Ga), while prosthetic groups are used rather than covalently binding the NMs with non-metallic radionuclides (e.g., ^11/14^ C, ^18^ F, ^76^Br, ^123^I, ^124^I, ^125^I, ^131^I) [112–127]. In the second strategy, radioisotopes are directly attached onto NMs by forming chemical bonds with nanomaterials. Pearson’s hard and soft acids and bases hypothesis stated that the soft acids (e.g., Ag, Au, Pt) react quicker and make strong bonds with soft bases (e.g., I, Se, Br), while hard acids (e.g., Zr, Y, Sr, Sc) react faster and produce stronger bonds with the hard bases (e.g., O, F) [111]. While using this method for radiolabeling, it should be noted that surface labeling must be approached carefully, as it may alter surface properties of NMs and, thus, affect their pharmacokinetics.

Inner incorporation is defined as the fabrication of imaging probes consisting of radionuclides integrated inside the nanomaterials. This strategy involves the use of two general approaches of incorporation of radioisotopic labels that can be either doped in the lattice of inorganic nanomaterials such as iron oxide and gold NPs, or been encapsulated into organic nanomaterials including liposomes or polymeric nanomaterials. In the first approach, radioactive agents are mixed with nonradioactive inorganic precursors, in both forms – aqueous and non-aqueous, to create nanoprobes with radioisotopes dipped in the crystal lattice of nanomaterials [128–130]. The second approach is based on incorporating of radionuclides either into the cavity of vesicle-like NPs, either with or without the addition of an additional ionophore and chelator to aid remote loading, or in the gallery space of core-shell nanostructures [131–133]. Specifically, radionuclides are attached onto the surface of NPs through the conventional surface binding technique followed by coating a protective layer of nanomaterials [134]. Apart from decoration of outer part with radionuclides or their loading into the inner space, they can also be embedded at the interface between nanomaterials and ligands. For example, diphosphate of PEG derivatives was simultaneously used as a chelating group for the radiotracer and as anchor group for surface PEGylation, allowing the radiotracer located between phosphate groups from neighboring PEG ligands [135].

Due to their unlimited penetration depth, whole-body capabilities, high sensitivity, quantifiable data, and the broad range of available radioisotopic labels, nuclear imaging modalities are highly suitable for the real-time monitoring of biodistribution profiles, pharmacokinetics, and desired site accumulation of the NMs in living subjects. Importantly, the high sensitivity of PET/SPECT imaging techniques enables visualization with sub-therapeutic doses of NMs in the microdose range. There are disadvantages associated with nuclear imaging approaches, however, including low spatial resolution, the lack of anatomical information, and the necessity to administer radioactive compounds. The first two can be overcome by co-scanning with anatomical imaging techniques such as CT or MRI. Multimodal imaging allows for the collecting of more detailed information on the overall levels of probe accumulation in the tissue or organ of interest due to the much clearer images of the anatomical and spatial distribution of the probe.

### Computed tomography

Computed tomography (CT) is a non-invasive imaging modality that takes advantage of differential tissue X-ray attenuation and thickness to produce cross-sectional and three-dimensional (3D) images of desired tissues and organs. The principle of this method is based on the ability to measure the density of the tissue passed by the X-ray beam by calculating the attenuation coefficient. CT uses the penetrating power of X-rays to produce a series of 2D radiographs of the subject viewed from different angles. Then the computed reconstruction algorithm creates a stack of cross-sectional slices from obtained 2D radiographs to provide 3D images of the internal structure of the subject [136]. CT is a commonly applied modality in clinical imaging and is traditionally used for hard tissue visualization of bone due to the high disparity between the soft and hard tissues. CT can easily differentiate tissues with high electron density tissues from relatively electron-poor structures [137]. Consequently, it is commonly used for orthopedic purposes, as well as for hybrid imaging applications, providing anatomical data with high resolution to aid in the evaluation of PET/SPECT- and FLI-based protocols [138, 139]. Its soft-tissue contrast, however, is quite inaccurate. Extrinsic contrast agents are required to obtain images of internal soft structures with high-resolution.

#### CT imaging probes

Historically, iodine was used as the atom of choice for the imaging applications of CT. There are two categories of iodine-based contrast materials: the ionic and nonionic agents. Although widely used in the clinics, the ionic iodinated imaging molecules have certain inherent drawbacks, such as the tendency to interact with biological structures and their increased intrinsic osmolality, which can potentially cause renal toxicity and other physiological issues. Moreover, high-osmolality contrast media causes reduced radio-density due to osmotic dilution. As opposed to the ionic iodinated contrasting agents, the nonionic counterparts have lower osmolality and display a low incidence of adverse health effects [140]. Multiple approaches have been explored in the fabrication of iodine-containing nanosized contrast agents, including liposomes, polymeric particles, micelles, nanospheres, nanosuspensions, etc. For example, iodinated small-molecule iohexol, has been widely exploited as CT contrast agent loaded into NMs for subsequent visualization and assessment of their biodistribution, circulation time, and tumor tissue accumulation [141, 142].

Another category of CT contrast agents are represented by metals with high X-ray attenuation coefficients (e.g., gold, barium, bismuth, tantalum, and zirconium). Gold NPs are one of the most commonly used metal-based radiopaque contrast agents since gold possesses both a high atomic number and a high density, resulting in favorable X-ray attenuating properties. Compared to iodine, gold generates 2.7 times more contrast per unit weight [143]. Gold NPs are highly suitable in evaluating the relationship between physicochemical properties of NPs and their in vivo performance. The gold NPs possesses some advantageous features, such as the ease of synthesis, good control over the size, ease of surface decoration with different biologically important ligands, high chemical stability, low toxicity and good biocompatibility [144]. Gold NPs can act as a standalone intravenous X-ray imaging agent to visualize their behavior in vivo, or they can be loaded into drug delivery systems to act as labeling agents for CT imaging.

## Nano-pharmaceutical evaluations under the bioimaging guidance

An understanding of the interactions between NMs and living subjects is extremely important. Studies describing the correlation between the structural and physicochemical properties of NMs (e.g., size, shape, surface properties, and drug release profiles) and their in vivo behavior can facilitate the establishment of new guidelines in accordance with the properties specific to nanomaterials, which may lead to faster and safer clinical translation. Moreover, the outcomes of these studies can provide a foundation for designing the next generation of novel NMs. What follows is a discussion of the influence that various key NMs parameters have on their in vivo fate, which has been monitored in a non-invasive and real-time manner.

### Size-dependent pharmaceutical evaluation of NMs under the bioimaging guidance

The complexity of determining the optimal size of NMs for a desired application necessitates additional investigations into the in vivo behavior of NMs. General nanoparticle design guidelines will emerge due to having better knowledge of the impact the size of NMs has on absorption, dispersion, permeability, and retention. Analytical models may guide the design and functionalization of NMs by providing a quantitative relationship between their size and functionality. In order to investigate the size-dependent in vivo behavior of NMs, numerous efforts have been made to develop imaging approaches with real-time monitoring.

**Fig. 4:**
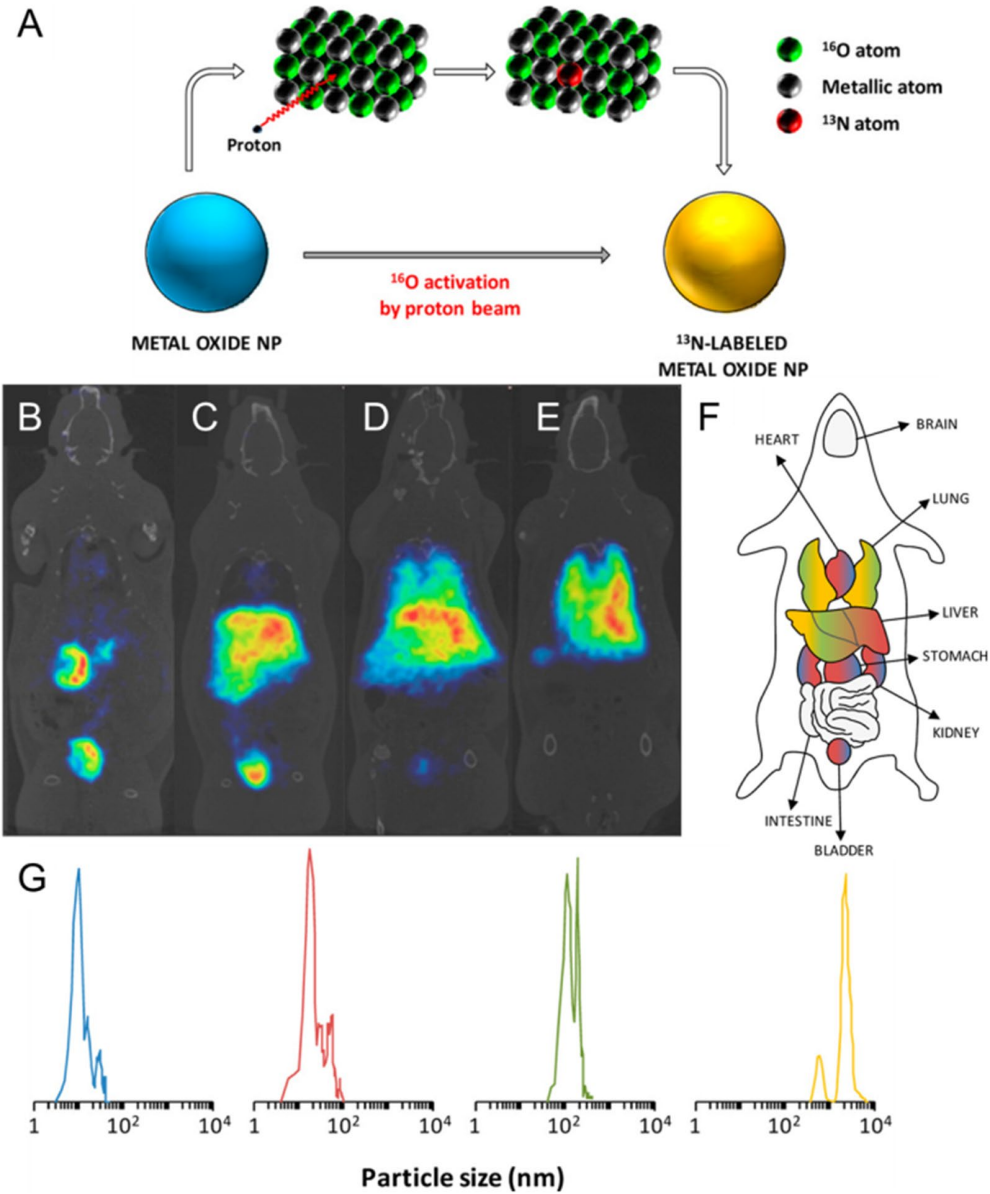
Size-dependent biodistribution of NMs. **A** activation of Al_2_O_3_ NPs by proton irradiation through the ^16^O(p,α)^13^ N nuclear reaction. Metal oxide NPs are activated with protons that convert ^16^O atoms to ^13^ N atoms by collision. B-E) PET visualization of _13_ N-labeled Al_2_O_3_ NPs signal at t_60_: NS_10nm_NPs **B**, NS_40nm_NPs **C**, NS_150nm_NPs **D**, and NS_10µm_NPs **E**. **F** particle size-dependent organ accumulation. **G** distribution of particle size evaluated by TEM (NS_10nm_,NS_40nm_,NS_150nm_) or DLS (NS_10µm_). Reproduced with permission from [153]. Copyright 2013, American Chemical Society

Circulation half-life is typically evaluation carried out by administrating the NMs and measuring their concentration in plasma at specified time intervals. In contrast, the non-invasive nature of biomedical imaging techniques realizes the direct and real-time monitoring of NMs’ pharmacokinetic behavior in vivo, without requiring the collection of blood samples [145, 146]. Detailed, non-invasive monitoring of the circulation profiles of NMs in blood vessels has attracted much interest in intravital imaging of the pharmacokinetic behavior of NMs in vivo. Biodistribution is intensely dependent on the hydrodynamic size of NMs and interactions with the living subjects. NPs with a hydrodynamic size less than 10 nm will be readily excreted by the kidneys, while NPs with a diameter larger than 200 nm will activate the complement system and will be rapidly cleared from the blood stream, accumulating in the liver and spleen [147–152]. Perez-Campana et al. developed activated, commercially available aluminum oxide (Al_2_O_3_) NPs with different sizes using direct irradiation with protons through the ^16^O(p,α)^13^ N nuclear reaction (Fig. 4A), exploiting them to perform real-time visualization of their biological fate using PET [153]. NPs are distributed throughout the vasculature, accumulating in various organs through distinct mechanisms in accordance with particle size. Large particles are captured by the smallest capillaries of the lungs or in the uptake of phagocytic cells in RES organs such as the liver, spleen, and lungs. Smaller particles accumulate in different organs by crossing the tight endothelial junctions and are quickly eliminated through the glomeruli of the kidneys (Fig. 4B–G).

Tumor tissue penetration and retention are another important parameter of NMs that is investigated while performing biodistribution studies. In contrast to free drug molecules, NMs accumulate in solid tumors more easily and selectively through the enhanced permeability and retention (EPR) effect, thus offering better antitumor activity [154]. The insufficient accumulation and inadequate tumor retention of NMs are generally attributed to sub-optimal particle sizes resulting in low therapeutic effects [155]. The application of in vivo imaging methods to assess tumor permeability and retention can greatly contribute to more rational NM designs that can achieve enhanced tumor-specific accumulation and retention to pursue efficient tumor eradication with fewer adverse effects. Permeability through the leaky tumor vasculature mainly depends on the size of both NMs and the pores. As NMs increase in size, the vascular permeability reduces [156]. The tumorous vascular pore size varies depending on tumor type and growth location [157]. For instance, Cabral et al. used a confocal laser scanning microscopy (CLSM) technique with a high-speed resonance scanner to intravitally evaluate the penetration and accumulation of the fluorescently labeled 30 and 70 nm micelles in C26 and BxPC3 tumors [158]. At 24 h post-injection (p.i.), the extravasated 30 and 70 nm micelles were detected inside the individual cells of C26 tumor tissue and their intensities were 40% of fluorescence intensity in the vasculature immediately after administration of the micelles. In BxPC3 tumors, the extravasation behavior of the 30 and 70 nm micelles was clearly different; the 30 nm micelles achieved deep tumor accumulation, while the 70 nm micelles remained close to the vasculature.

**Fig. 5:**
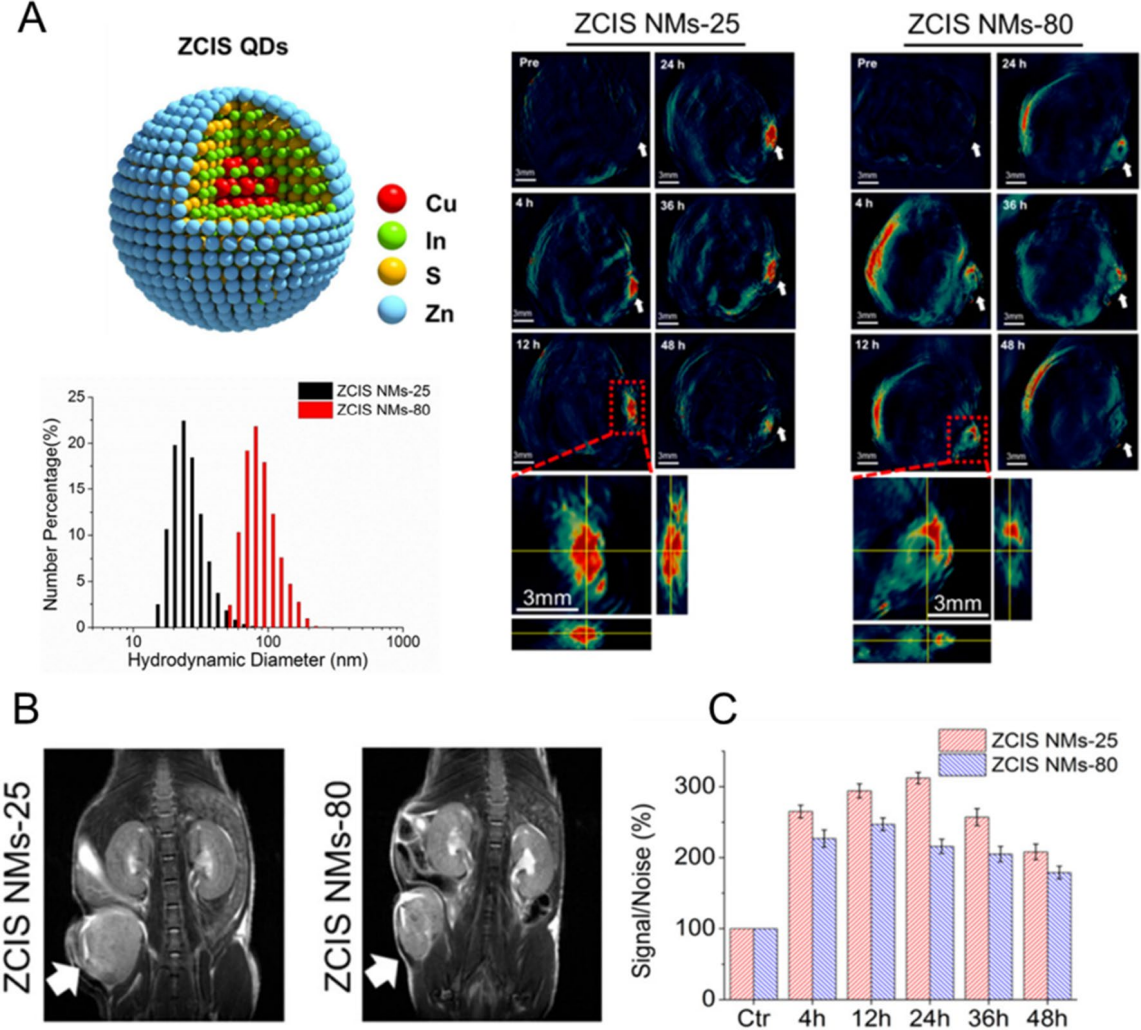
Size-dependent intratumoral accumulation of NMs. **A** Left: schematic illustration of ZCIS QDs and hydrodynamic diameters distribution of ZCIS NMs-25 and ZCIS NMs-80. Right: in vivo multispectral optical tomography (MSOT) images of tumors (indicated with white arrows) in mice obtained at different time points after i.v. injection of ZCIS NMs-25 and ZCIS NMs-80. **B** In vivo MRI images of the tumor bearing mice obtained at 48 h p.i. (arrows indicate tumors). **C** MSOT signal increase in the tumor at various times p.i. Reproduced with permission from [159]. Copyright 2016, American Chemical Society

In another study, Lv et al. fabricated lipid (DSPE-PEG-2 K)-coated CuInS/ZnS quantum dots (ZCIS QDs) with 25 and 80 nm hydrodynamic sizes [159]. They found that the ZCIS QDs with a hydrodynamic size of 25 nm offer prolonged tumor retention time, greater tumor uptake, and deeper 4T1 tumor penetration compared to ZCIS QDs with a hydrodynamic size of 80 nm (Fig. 5). Similarly, Popovoc et al. also prepared a size series of luminescent, water-soluble QDs within the 10–150 nm size range, employing them in the study of the real-time, size-dependent particle transport parameters in solid tumors [160]. A mixture of 12 nm, 60 nm, and 125 nm NPs with various emission wavelengths were simultaneously administrated into a mouse bearing a Mu89 human melanoma, followed by intravital multiphoton microscopy of several regions of the tumor. It was observed that the 12 nm NPs extravasated easily, albeit heterogeneously, diffusing from the vessels with minimal hindrance. In contrast, the 60 nm NPs did extravasate; they did not, however, leave the immediate perivascular space, remaining within 10 μm of the vessel walls. The 125 nm particles did not noticeably extravasate.

**Fig. 6:**
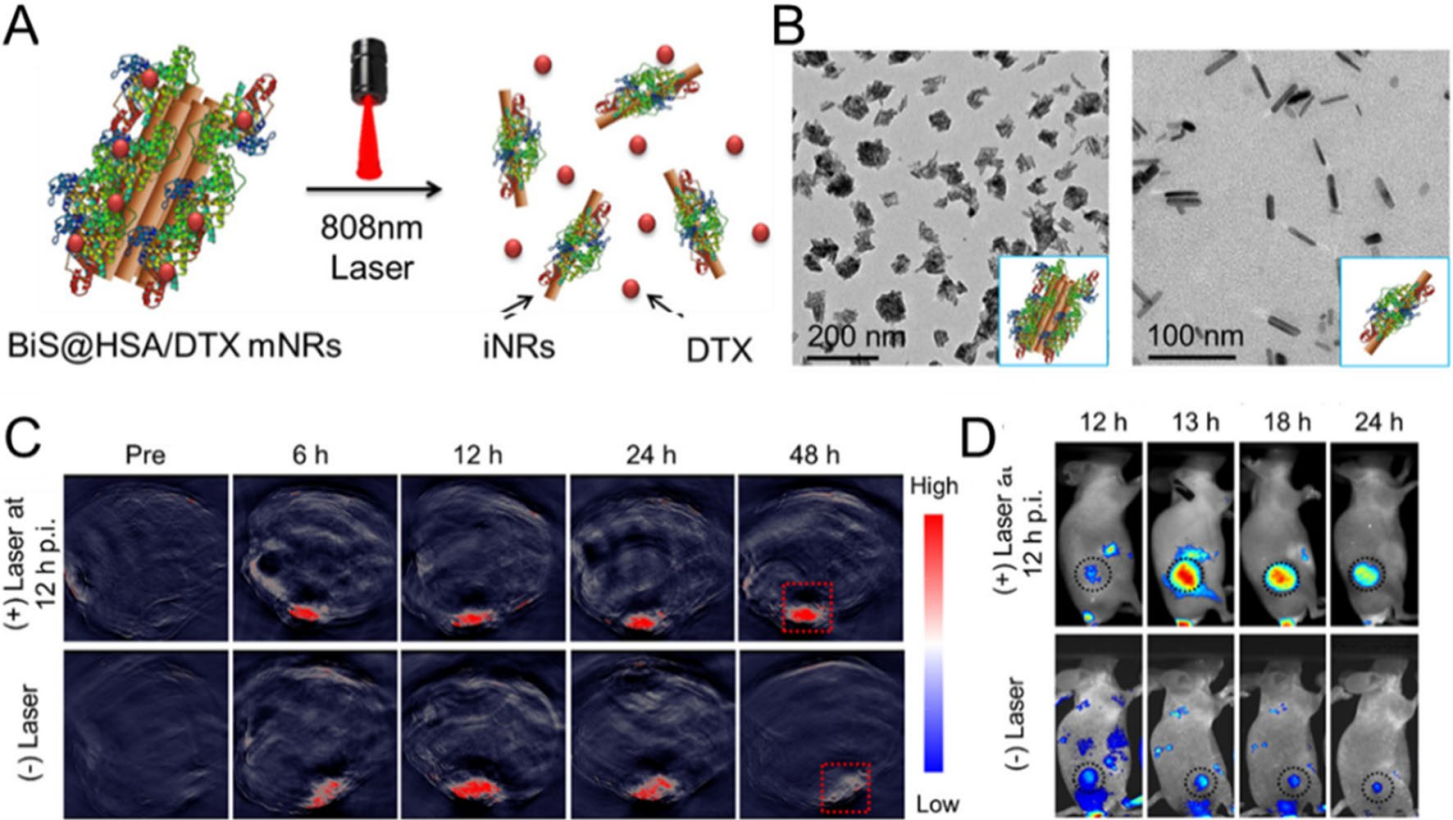
Size-dependent intratumoral behavior of NMs. **A** schematic illustration of the disassembly of BiS@HSA/DTX mNRs and DTX release upon laser exposure. **B** TEM images and schematic pictures (insets) of BiS@HSA/DTX mNRs before (left) and after (right) laser irradiation (808 nm, 1 W/cm^2^, 10 min). **C** PA visualization of tumors in mice with or without laser irradiation at 12 h p.i. **D** fluorescence images of tumor-bearing mice obtained at various time points with or without laser exposure. Reproduced with permission from [161]. Copyright 2018, American Chemical Society

**Fig. 7:**
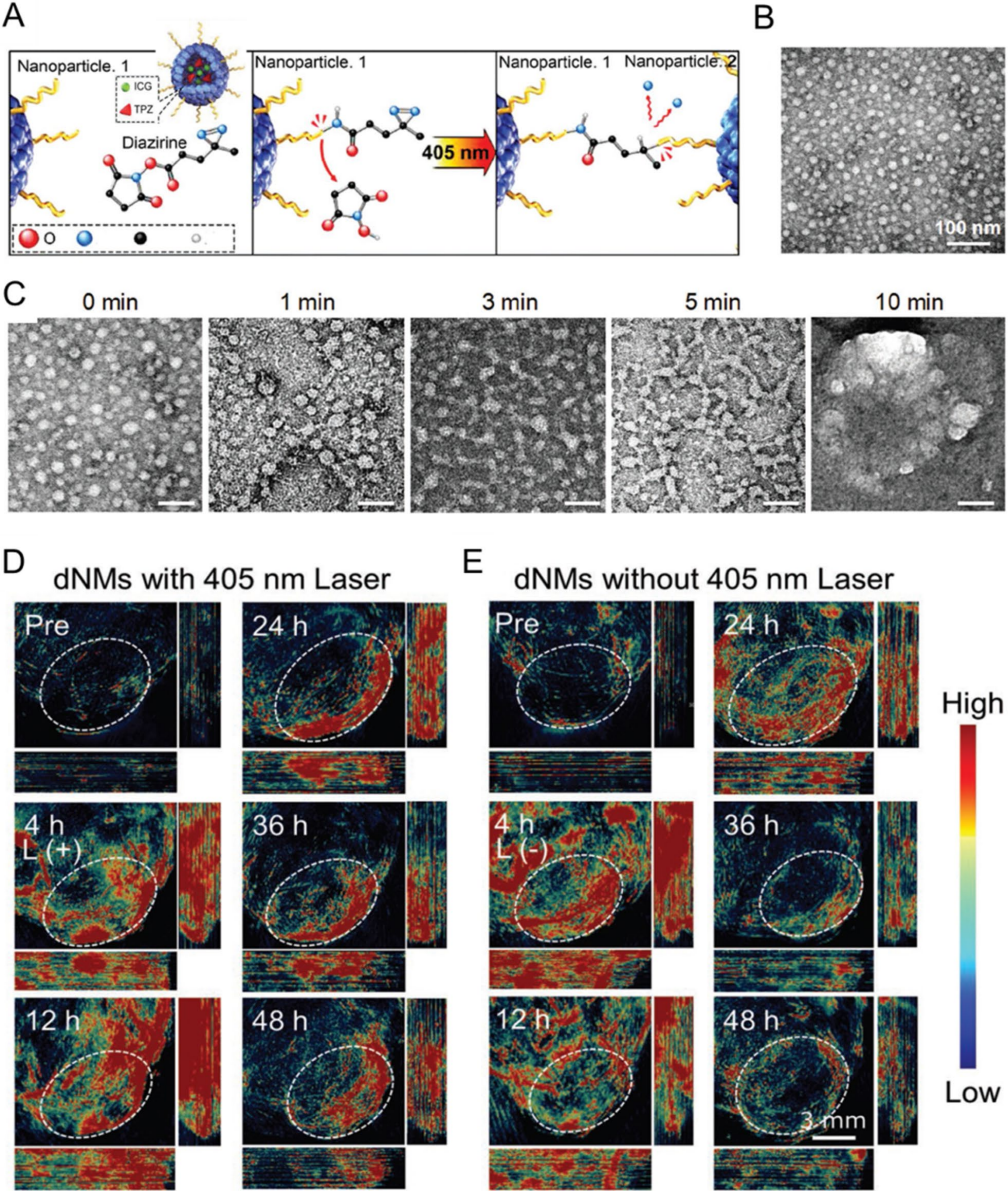
Size-dependent intratumoral persistence of NMs. **A** schematic representation of laser-induced aggregation of dNMs. **B** TEM image of ICG/TPZ@HSA dNMs (scale bar: 50 nm). **C** TEM images of dNMs at different time points upon laser irradiation. **D** PA visualization of dNMs in 4T1 tumor-bearing mice following i.v. administration with. **E** PA visualization of dNMs in 4T1 tumor-bearing mice following i.v. administration without laser irradiation at different time points. Reproduced with permission from [167]. Copyright 2018, Wiley-VCH

Researchers have developed different size-adjustable NMs with greater initial sizes during blood circulation for slower elimination, converting into smaller particles in the tumor area induced by internal or external stimuli for efficient extravasation from tumor vessels, better intratumoral distribution, and longer retention [162–165]. For instance, Guo and colleagues employed PA/CT dual-modal imaging to monitor intratumoral behavior of NIR laser-triggered transformable multiple nanorods (mNRs) in a real-time and non-invasive manner [161]. The fabricated mNRs consisted of small bundles of uniform bismuth sulfide (BiS) NRs that were bound together by human serum albumin (HSA), with Docetaxel (DTX) entrapped in the HSA corona (BiS@HSA/DTX mNRs). These NMs exhibited an initial size of 100 nm and rapidly disassembled into smaller individual NRs (BiS@HSA iNRs) with a size of 40 nm under NIR laser exposure due to the photothermal properties of BiS NRs. After systemic administration in nude mice bearing MDA-MB-231 breast cancer, the NMs not only showed effective extravasation from the blood vessels and accumulation in the tumor tissue *via* the favorable EPR effect, but also demonstrated better intratumoral distribution and prolonged retention by taking advantage of the diffusion superiority of smaller NPs (Fig. 6). Indeed, smaller particles penetrate more deeply into the tumor tissue; they can still suffer, however, from inadequate accumulation and retention caused by dynamic equilibrium between the infiltration and extravasation of the NMs through the leaky tumor vasculature [158, 166]. Chen et al. proposed an opposite in vivo size manipulation strategy that can also be applied to ameliorate the intra-tumor trafficking profile of systemically administered nanomedical drugs [167]. Their research group fabricated a sophisticated system of human serum albumin (HSA) NMs decorated with diazirine (DA) and co-loaded with the photo-sensitizer indocyanine green (ICG) and the activatable chemo-drug tirapazamine (TPZ). ICG/TPZ@HSA dNMs were sensitive to successive laser irradiations and exhibited the crosslink and enlargement of the particles triggered by laser exposure. The recorded fluorescence images of 4T1 tumor-bearing mice demonstrated that the large size of dNMs – caused by laser exposure–prevented their re-penetration into blood vessels, resulting in greater tumor tissue accumulation and longer-term intratumoral persistence as compared to the individual NPs (Fig. 7).

### Shape-dependent pharmaceutical evaluation of NMs under the bioimaging guidance

**Fig. 8:**
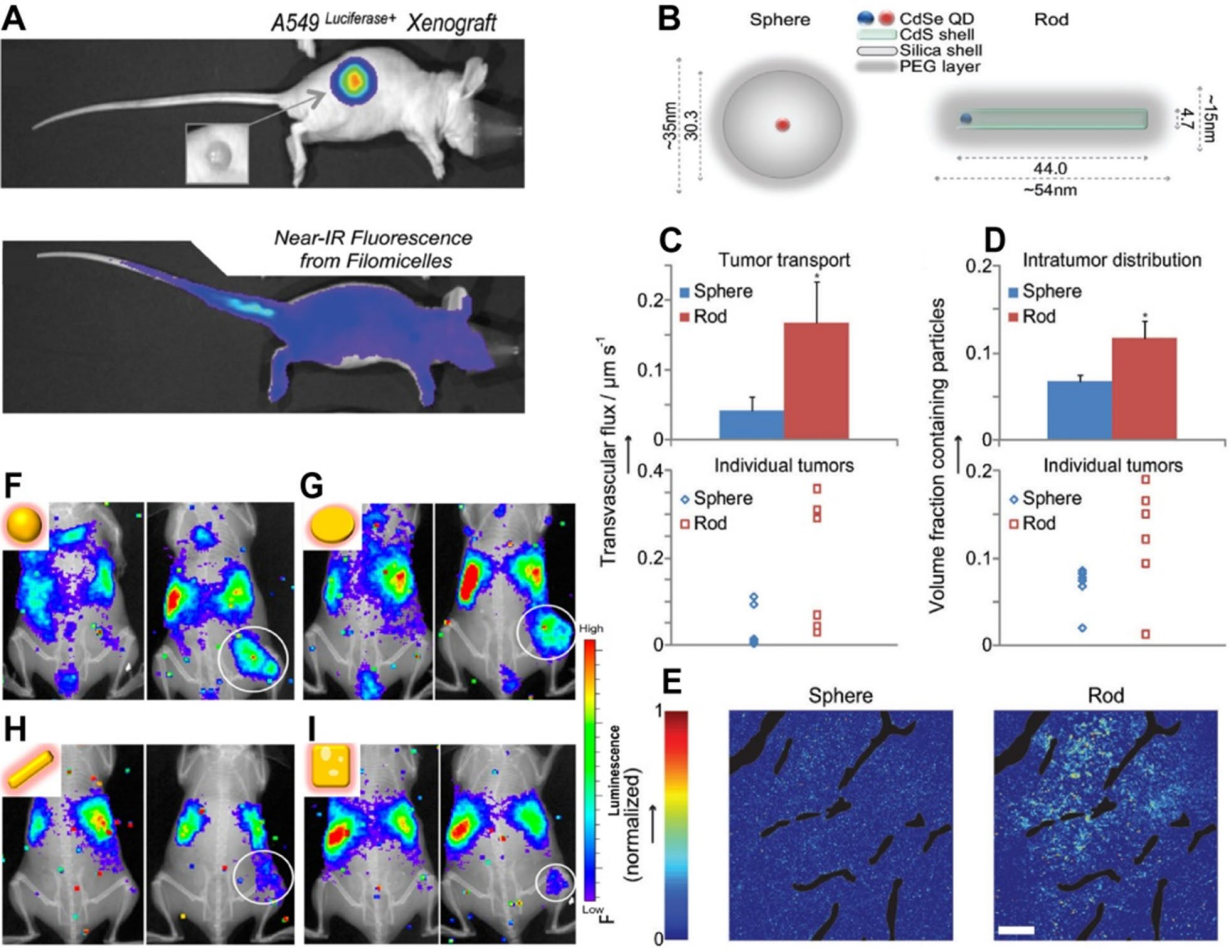
Shape-dependent biodistribution of NMs. Biodistribution of filomicelles in A549 tumor xenograft mice: **A** upper panel: whole body bioluminescent images of luciferase-transfected A549 tumor xenograft mice (inset: photograph of tumor); lower panel NIR fluorescence image of A549 tumor xenograft mouse that shows the diffuse fluorescence of NIRF-labeled filomicelles in circulation. Transport and distribution in tumors in vivo for nanospheres versus nanorods of the same hydrodynamic diameter. **B** schematic illustration of nanospheres and nanorods. **C** Transvascular transport rates and **D** distribution in orthotopic E0771 mammary tumors in vivo for nanospheres versus nanorods of the same hydrodynamic diameter (33–35 nm); **E** NPs penetration in tumors. Co-registered in vivo luminescence and X-ray images of the tumor-bearing mice at 1 h (left panel) and 24 h (right panel) p.i. of the various types of ^198^Au-incorporated nanostructures: **F** nanospheres, **G** nanodisks, **H** nanorods, and **I** cubic nanocages. Reproduced with permission from [179, 181, 182]. Copyright 2009, 2014, American Chemical Society; 2011, Wiley-VCH

The shape of NMs represents another essential parameter that determines their effect on biological processes associated with therapeutic delivery. Although most of the NMs currently undergoing preclinical or clinical studies have spherical shapes, the unique features of non-spherical NMs may provide new avenues for rationally designing NMs for specific applications. Recent researches have revealed that macrophage uptake is strongly influenced by the shape of NMs [168–171]. It was demonstrated that prolate ellipsoids have high attachment ability but the lowest internalization, whereas oblate ellipsoids displayed both strengthened attachment and strengthened internalization, resulting in the highest phagocytosis [172]. Along with phagocytosis rates, the hydrodynamic behavior of NMs in the blood flow also affects their circulation time. It was revealed that non-spherical NPs – particularly nanorods, nanodiscs, and nano worms – exhibited deviating hydrodynamic behavior with respect to their spherical counterparts, allowing them to align with the blood flow, which led to the extension of the circulation time [173–178]. Christian et al. applied in vivo real-time imaging to track the biodistribution and organ accumulation of paclitaxel-loaded filomicelles labeled with near-infrared fluorophores (NIRFs) [179]. Filomicelles exhibited delay in the rapid clearance by the RES, remaining in blood circulation for a minimum of 24 h after intravenous injection (Fig. 8A, B). In comparison to the identical spherical micelles, filomicelles displayed greater tumor shrinkage and less apoptosis in non-tumor tissues.

The shape of NMs also influences their biodistribution profiles. The different shapes have also been revealed to impact in vivo distribution patterns, providing new strategies for targeting desired organs. Cylindrical NPs have displayed the greatest accumulation in the liver compared to their spherical, hemispherical, and discoidal counterparts [180]. In contrast, discoidal NPs accumulated more in vascular organs such as spleens and lungs. Hemispherical NPs demonstrated similar distribution behavior to the spherical NPs, except they exhibit high spleen accumulation but a lesser degree of accumulation in the lungs. Minor alterations in particle shape may also substantially mediate the biodistribution of NMs. For instance, NPs of similar shape but with various aspect ratios have significantly different organ biodistributions [175]. Short-rod mesoporous silica NPs (MSNs) were shown to be easily retained in the liver, while long-rod NPs accumulated in the spleen. PEGylation led to higher distribution in the lungs for both types of NPs, and more elevated accumulation was detected for the long-rod NPs. The clearance rate of MSNs was found to be predominantly influenced by particle shape, where short-rod MSNs showed a more rapid clearance rate compared with long-rod MSNs excreted by urine and feces.

In addition to systemic circulation and biodistribution, the shape of the NMs can also influence the targeting the vascular endothelium and disease sites. Non-spherical NPs exhibit distinctive tumbling and rolling motions that result in diverse margination dynamics. Elongated particles usually have a lower drag coefficient and a greater surface area than spheres [183, 184]. Thereby, certain non-spherical NPs can adhere to the endothelial walls with greater strength because of increased multivalent bonding. Moreover, non-spherical NPs were found to possess higher degrees of lateral drifting and stronger adhesion to vessel walls, providing them with a better chance to interact with the endothelium [185–187]. The geometry of NMs was revealed to modulate disease targeting and internalization processes as well. NMs of certain non-spherical shapes demonstrated increased tumor targeting and accumulation due to factors such as prolonged circulation time, higher margination, adhesion, and extravasation; they also demonstrate less retrieval of NPs from the tumor microenvironment [188–193]. For example, Chauhan et al. applied real-time in vivo imaging to demonstrate that quantum dot-based nanorods possessed more rapid intratumoral penetration relative to quantum dot-based nanospheres (Fig. 8C–E) [181]. Similar results were obtained for gold NPs, where gold nanorods accumulated more markedly in tumor tissues than gold nanospheres in an orthotopic A2780 human ovarian cancer model [178]. In addition, radioactive ^198^Au-loaded gold nanocages of spherical shape as well as nanodiscs were found to have distinctive tumor uptake profiles compared to nanorods and cubic nanocages (Fig. 8F–I) [182]. In vivo luminescence visualization was conducted on the mice bearing EMT6 tumors by monitoring the Cerenkov radiation with a conventional optical in vivo imaging system. The nanospheres exhibited the longest half-life circulation, the lowest elimination by the RES, and the greatest tumor accumulation compared to nanorods, nanocages, and nanodiscs. Interestingly, nanorods and nanocages were observed in the tumors’ cores, while nanospheres and nanodiscs were distributed only on the tumors’ surfaces.

### Surface-dependent pharmaceutical evaluation of NMs under the bioimaging guidance

Surface chemistry is one of the critical parameters of the in vivo behavior of NMs and encompasses such properties as surface hydrophobicity, surface charge, and targeting ligands, amongst others. Surface features directly determine the interaction between NMs and physiological environments; hence, they substantially influence the in vivo behavior of NMs by affecting various processes like the formation of a protein corona, systemic circulation time, biodistribution, cellular uptake, and clearance from the body [194, 195]. There is a growing focus on manipulating and optimizing the surface properties of NMs in order to increase in vivo therapeutic index while reducing adverse effects. Non-invasive imaging techniques can be integrated at this stage of the NMs development pipeline since the understanding of the intrinsic connection of the relationship between surface chemistry and in vivo behavior remains one of the pressing priorities in the progress of NMs.

#### Surface charge

**Fig. 9:**
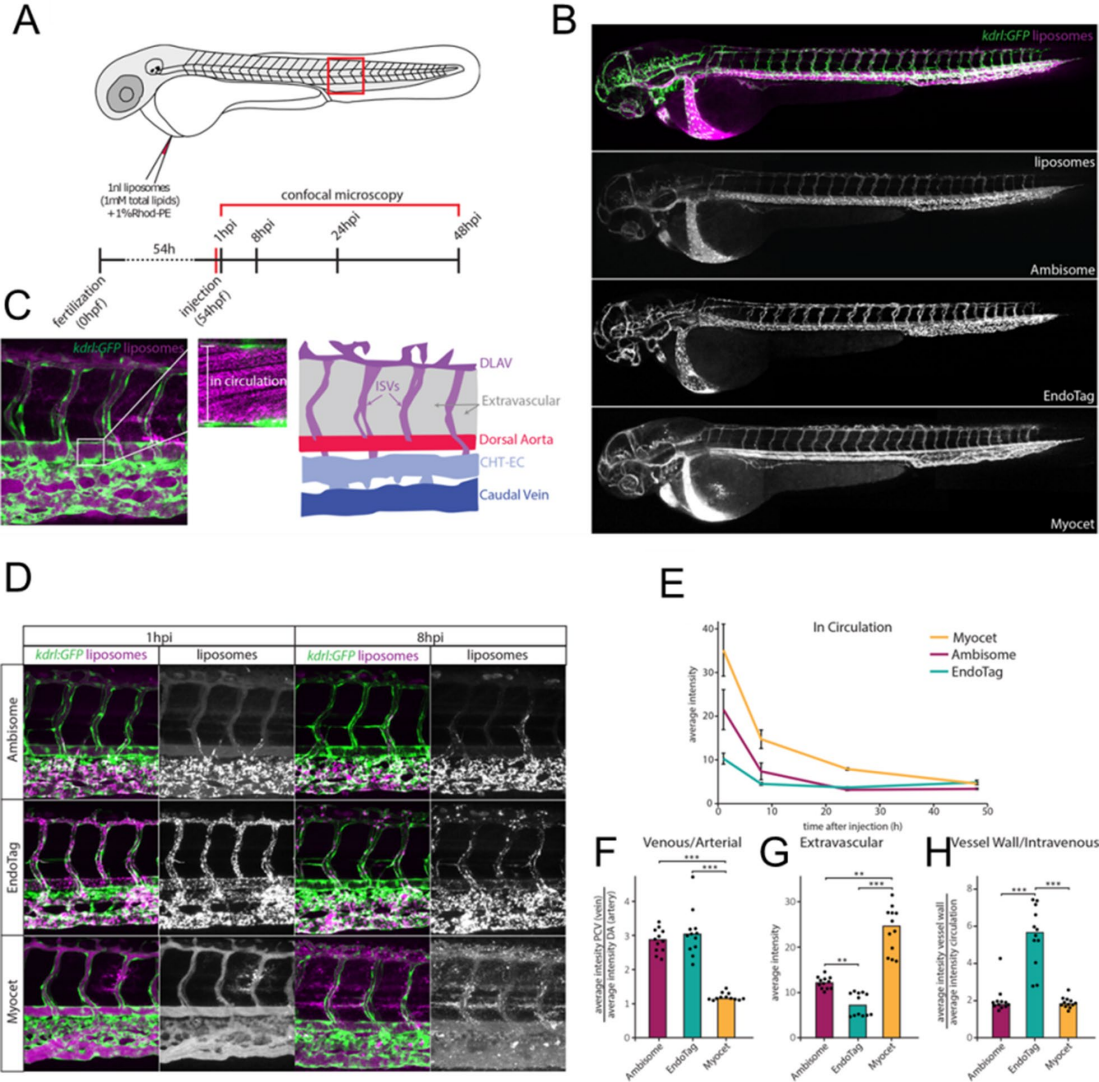
Surface charge-dependent biodistribution of NMs. **A** schematic of illustration of fluorescently labelled liposomes and quantification in zebrafish. **B** liposomes distribution in *kdrl:GFP* transgenic embryos, 1 h p.i. **C** in vivo imaging of liposomes in circulation (measured in the lumen of the dorsal aorta, and liposome association with different blood vessel types. CHT-EC: caudal hematopoietic tissue endothelial cells, DLAV: dorsal longitudinal anastomotic vessel. ISV: intersegmental vessel. **D** liposomes distribution at tissue level. Quantification of liposome levels: **E** in circulation based on mean rhodamine fluorescence intensity in the lumen of the dorsal aorta. **F** associated with venous vs. arterial endothelial cells based on rhodamine fluorescence intensity associated with caudal vein (CV) vs. DA at 8 h p.i. **G** outside of the vasculature between the DLAV and DA at 8 h p.i. **H** associated with the vessel wall based on rhodamine fluorescence intensity associated with all endothelial cells relative to rhodamine fluorescence intensity in circulation at 1 h p.i. Reproduced with permission from [205]. Copyright 2018, American Chemical Society

Extensive studies have demonstrated that high surface charge densities–either anionic or cationic–usually cause rapid blood clearance and uptake by RES, whereas neutral charges lead to prolonged half-life and decreased RES clearance [196–199]. For instance, it was shown that fabricated PEG-oligocholic acid NPs with surface charges in the range of − 26.9 to 37.0 mV were phagocytosed in a higher degree if the surface charge was highly positive (> 15 mV) or negative (< − 15 mV), while NPs with slightly negative charges (− 8.5 mV) exhibited lower Kupffer cell uptake and accumulation in the liver [200]. In another study, chitosan-grafted polymeric NPs displayed similar surface charge-dependent in vivo performance [148]. Cationic NPs had a shorter half-life and greater liver and spleen accumulation compared to anionic NPs, whereas higher negative charge densities caused enhanced liver accumulation and less retention in the blood. In addition, positively charged NPs tend to adsorb into the anionic surface of cells and are subsequently internalized, often resulting in acute cytotoxicity such as hemolysis and platelet aggregation [201–204]. Given the unfavorable pharmacokinetics and biodistribution of charged NPs in the body, the most clinically approved NMs have neutral (or near-neutral) surface charge [12]. Campbell et al. used real-time fluorescence imaging to compare differently charged liposomes that are either approved for clinical use or under development (EndoTAG-1, Myocet, and AmBisome) [205]. The IV-administered liposomes with a hydrodynamic size of 100 nm and neutral surface charge tended to freely circulate in embryonic zebrafish, while negatively charged NPs (< − 20 mV) interacted strongly with RES cell types, particularly scavenging endothelial cells (astabilin-mediated clearance pathway) and blood resident macrophages. The liposomes bearing positive charge (> 20 mV) were rapidly cleared from circulation through both non-specific cellular interactions and clearance *via* the RES (Fig. 9).

**Fig. 10:**
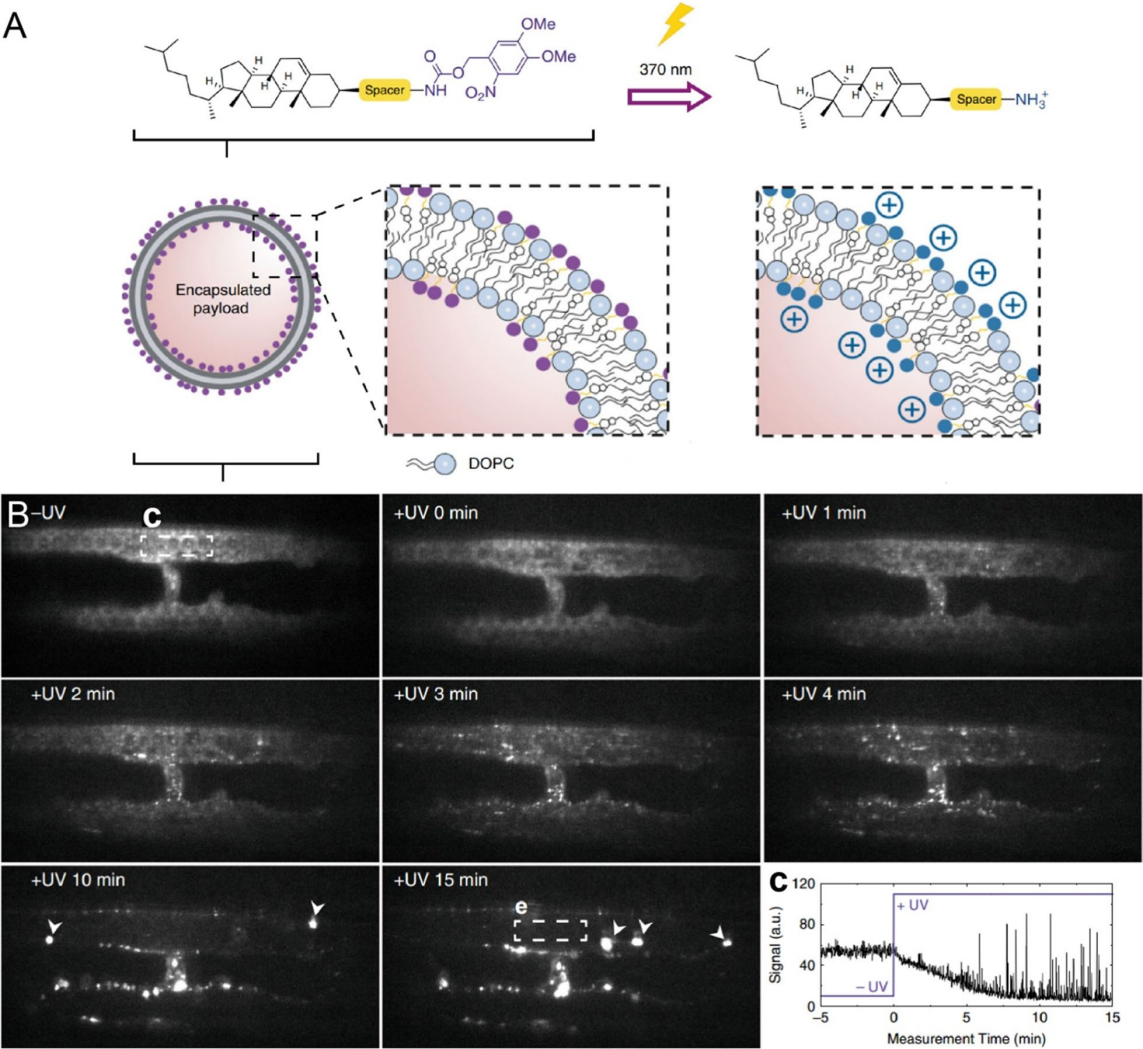
Surface charge-dependent circulation of NMs. **A** schematic representation of photoswitching of the surface charge of a liposome. **B** real time multi-photon imaging of liposome distribution with or without UV exposure. At later times, large aggregations of liposomes (white arrows) were detected passing through the plane of view in circulation. **c** mean fluorescence intensity within the lumen of dorsal aorta (white square, − UV and + UV 15 min). Fluorescence intensity of liposomes immediately reduced upon UV exposure. Large circulating aggregates of liposomes caused high intensity spikes of fluorescence registered after 5 min of UV irradiation. Reproduced with permission from [211]. Copyright 2020, Springer Nature

Surface charge also plays a substantial role in the accumulation of NMs in the affected areas. The accumulation of NMs in tumors depends on systemic circulation and interaction with tumor-associated cells that are influenced by surface charge [195, 206, 207]. NMs with a positive charge have been demonstrated to possess enhanced preferential adhesion and permeability in angiogenic tumor vessels with respect to their anionic or neutral counterparts and normal vasculature [208, 209]. In addition, positively charged surfaces facilitate non-specific adsorption and tumor cellular uptake as well as promoting endosomal escape and cargo release in cytosol. In contrast, negatively charged and neutral NMs are poorly internalized by non-RES cell lines, and those that are taken up tend to localize within lysosomes [203, 210]. Thus, on the one hand, neutral NMs circulate freely, ensuring high biodistribution throughout the body; on the other hand, cationic NMs are non-specifically taken up by almost all cells, leading to poor pharmacokinetics, while providing high intracellular concentrations of encapsulated cargos. In this regard, it is a promising strategy to develop NMs with switchable surface charges to achieve desired in vivo behavior and improved therapeutic efficacy. For example, Arias-Alpizar et al. designed photoactive liposomes with surface charges that are able to rapidly switch from neutral to cationic, *in situ*, and in vivo in response to light irradiation [211]. Small and transparent zebrafish embryos were selected as the model organism to image NMs across whole live organisms at cellular resolution and in real time. The results have shown that, prior to light exposure, liposomes freely circulated and did not noticeably interact with RES or other cell types. The rapid surface charge switching caused by *in situ* light irradiation resulted in not only non-specific adsorption and uptake of liposomes across the entire endothelium of the zebrafish embryo, but also in uptake by phagocytes in blood-resident macrophages (Fig. 10).

In another study, Hung and colleagues developed a nanovehicle system comprising poly(lactic-co-glycolic acid) (PLGA) as the hydrophobic cores coated with pH-responsive N-acetyl histidine modified D-α-tocopheryl polyethylene glycol succinate (NAcHis-TPGS) [206]. It was demonstrated that these NMs were able to change surface charge from negative to nearly neutral or slightly positive values in response to a decrease of pH that further had a positive impact on their biodistribution in mice bearing TRAMP-C1 tumor model. In vivo real-time observations revealed that after intravenous injection the NMs could significantly accumulate within solid tumor because of their pH-triggered near neutral surfaces.

#### PEGylation

**Fig. 11:**
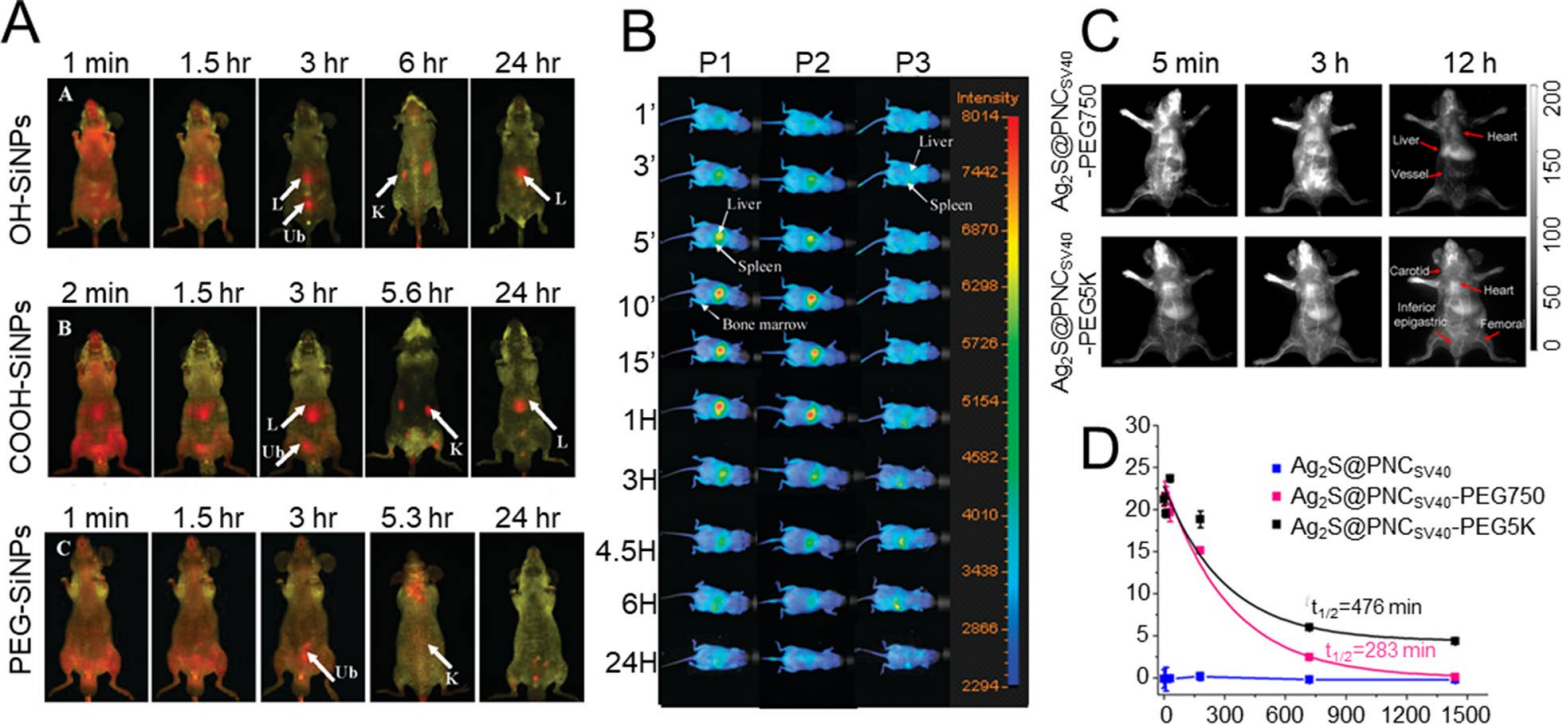
PEG length-dependent biodistribution of NMs. **A** in vivo visualization of various surface-modified SiNPs biodistribution at various times (a - abdomen imaging, b - back imaging). Arrows indicate kidney (K), liver (L), and urinary bladder (Ub) location. Reproduced with permission from. **B** time-dependent in vivo imaging of ITK705-amino QDs coated with methoxy-terminated PEG of different chain length: P1 = 2.75 kDa, P3 = 7.0 kDa, P5 = 22 kDa. Reproduced with permission from. **C** time course of fluorescence images of mice treated with PEGylated Ag_2_S@PNC_SV40_. **D** blood circulation curves of uncoated and PEGylated Ag_2_S@PNC_SV40_ in mice. Reproduced with permission from [214–216]. Copyright 2008, 2009, 2015, American Chemical Society

NMs with a hydrophilic surface are able to resist plasma protein adsorption and avoid uptake by macrophages and RES clearance. This can be achieved by either coating the surface with hydrophilic polymers/moieties such as PEG and its analogues (poloxamer and poloxamine series), dextran, and chitosan, or by directly formulating NMs from block copolymers consisting of both hydrophilic and hydrophobic segments [212, 213]. He et al. fabricated three types of surface-modified silica NPs (SiNPs), including OH-SiNPs, COOH-SiNPs, and PEG-SiNPs with a size of ∼ 45 nm and loaded with a RuBPY dye with the purpose of studying biodistribution and urinary excretion by exploiting the in vivo fluorescence imaging system [214]. The study’s outcome revealed that the PEG-SiNPs displayed a substantially longer circulation half-life (t_1/2_=180 ± 40 min) compared to OH-SiNPs (t_1/2_=80 ± 30 min) and COOH-SiNPs (t_1/2_=35 ± 10 min). Moreover, the in vivo imaging results also demonstrated that all the IV-injected types of SiNPs were partly cleared *via* the renal route (Fig. 11A). In another study, Xiao and colleagues applied in vivo real-time FLI and PA imaging to demonstrate that the modification of the surface of self-assembled hydrophobic NIR dye IR-797 NPs with amphiphilic polymer C18PMH-PEG5000 (PEG-IR-797) could significantly increase accumulation and retention of NPs in tumor due to prolonged circulation time and EPR effect [82]. Naked IR-797 NPs were used as a control and displayed only moderate accumulation in tumor tissue 12 h after injection. Further biodistribution analysis revealed increased fluorescence intensities of PEG-IR-797 NPs in liver and spleen, while naked IR-797 were demonstrated to be quickly eliminated through the kidney.

**Fig. 12:**
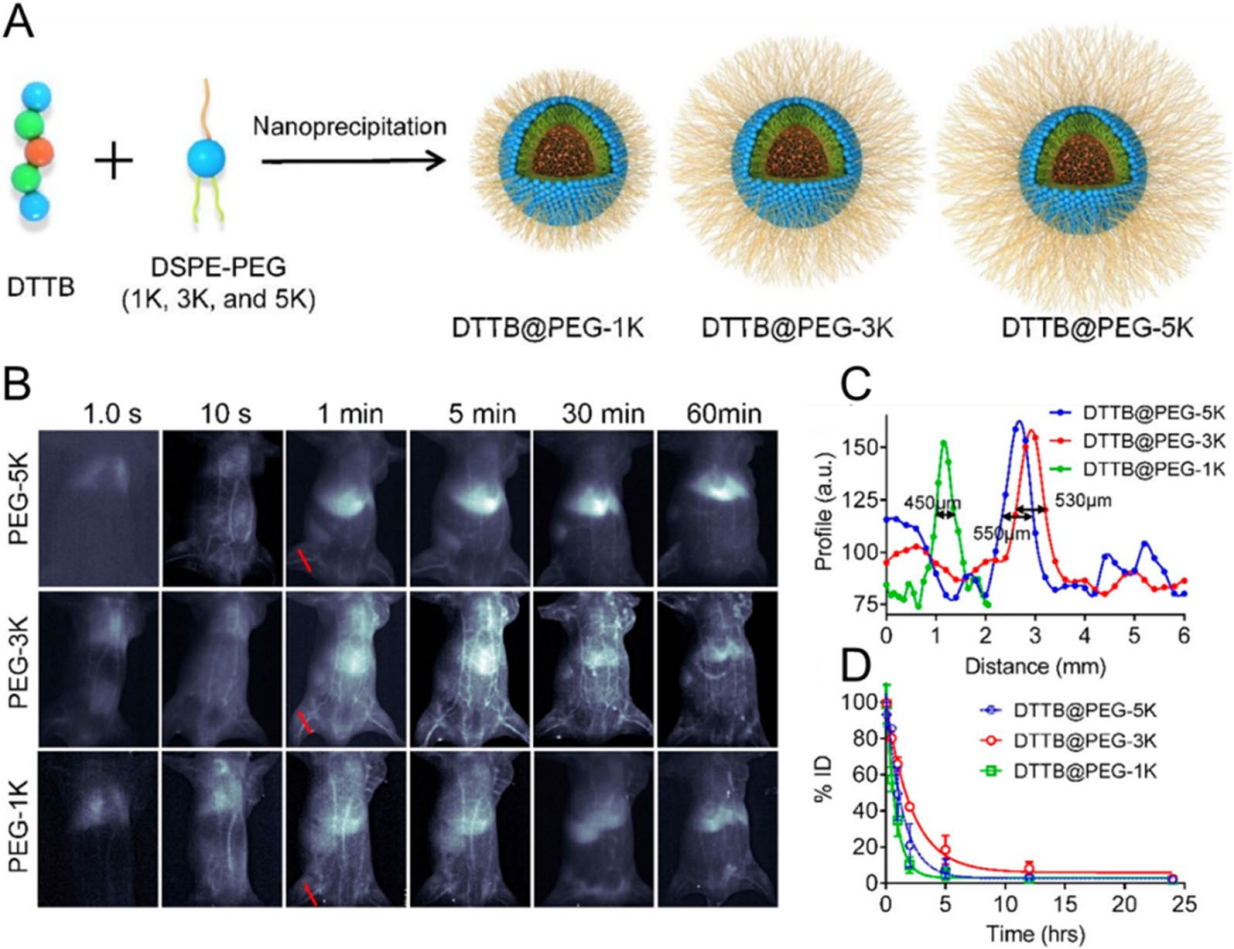
PEG length-dependent circulation of NMs. **A** schematic illustration of DTTB@PEG NMs preparation. **B** real-time intrinsic NIR-II visualization of whole-body vascular network on nude mice recorded 60 min p.i. **C** vessel FWHM width analyses (red lines in panel A) based on the cross-sectional intensity profiles. **D** pharmacokinetics of NMs following i.v. administration. Reproduced with permission from [218]. Copyright 2020, American Chemical Society

The impact made by the length and density of PEG chains on the intrinsic behavior of NMs has been widely investigated. Various reports suggest that higher density and longer PEG length have better chances to mask the nanocarrier charge or surface hydrophobicity [195]. Daou et al. fabricated commercial ITK705-amino QDs coated with methoxy-terminated PEG of various chain lengths (2.75–22 kDa) [215]. The effect of the particle coating on their intrinsic behavior after IV administration was monitored using non-invasive fluorescence imaging. Fluorescence from all QDs was easily observed after injection in the superficial vasculature, which was followed by the detection of QDs in the liver, spleen, bone marrow, and skin in a PEG length-dependent manner (Fig. 11B). The slower accumulation in the liver corroborated with a prolonged half-life of coated QDs. Thus, an increase of PEG length seems rather beneficial for reducing RES uptake, as more goes along with a neutralization of the negative charges of the QDs. In another study, protein nanocages (PNCs) were decorated with PEG of different lengths (0.75 and 5 kDa) and loaded with Ag_2_S QDs (Ag_2_S@PNC_SV40_-PEG750 and Ag_2_S@PNC_SV40_-PEG5K) for real-time tracking of NP migration and biodistribution [216]. Real-time NIR-II fluorescence imaging first displayed migration of both Ag_2_S@PNC_SV40_-PEG750 and Ag_2_S@PNC_SV40_-PEG5K to the heart and lung immediately after IV injection, followed by distribution into the whole body *via* the systemic circulation. Unlike the naked Ag_2_S@PNC_SV40_, the PEGylated PNC_SV40_ demonstrated significantly enhanced blood retention with detectable fluorescence signals persisting over 12 h. In contrast, Ag_2_S@PNC_SV40_-PEG5K showed negligible fluorescence detected in the RES system. Further quantitative assessments confirmed rapid clearance of uncoated PNCs within five minutes p.i., while Ag_2_S@PNC_SV40_-PEG750 and Ag_2_S@PNC_SV40_-PEG5K displayed substantially extended blood circulation times of 283 and 476 min, respectively. Further evaluation of half-lives by ICP-MS agreed well with the various fluorescence profiles of naked and PEGylated Ag_2_S@PNC_SV40_ in living mice, supporting the validity of the outcomes obtained by NIR-II imaging (Fig. 11C,D). Similarly, Khargharia constructed polyacridine peptide nanocarriers with different PEG lengths (2, 5, 10, 20, or 30 kDa) and investigated their in vivo performance, particularly in terms of pharmacokinetic profiles and biodistribution [217]. The outcomes of this study revealed that an increase of PEG length leads to circulation time extension and reduction of liver accumulation. It should be noted, however, that the dogma “longer is always better” does not apply to all cases. To confirm this, Li et al. have constructed a high-emissive NIR-II luminophore based on alternative donor-acceptor-donor (D-A-D) conjugated oligomer (DTTB), binding it with PEG ligands at different chain lengths (DTTB@PEG-1 K, DTTB@PEG-3 K, and DTTB@PEG-5 K) [218]. The sharpness feature imaged in the NIR-II window for DTTB@PEG-1 K, DTTB@PEG-3 K, and DTTB@PEG-5 K was 450, 530, and 550 μm, respectively (Fig. 12C). It was observed that the vasculature of DTTB@PEG-3 K NM-injected mice was still clearly visible one hour p.i., whereas distinct signals were detected in the liver and spleen of the mice that were exposed to DTTB@PEG-1 K and DTTB@PEG-5 K NMs (Fig. 12B). This suggested prolonged half-life of DTTB@PEG-3 K NMs in comparison to DTTB@PEG-1 K and DTTB@PEG-5 K NMs. The outcomes of real-time evaluations of circulation lifetimes were further confirmed by pharmacokinetic measurements that displayed the longest elimination half-life of DTTB@PEG-3 K NMs (1.49 h) with respect to DTTB@PEG-1 K (0.54 h) and DTTB@PEG-5 K NMs (0.95 h) (Fig. 12D).

Similarly, PEG density also impacts the intrinsic behavior of NMs by having a greater effect on the coating of the surface of a nanocarrier and its accessibility to plasma proteins [219]. For example, Barratt et al. suggested that the enhancement of the PEG density of nanocarriers from 10 to 30% resulted in a remarkable decrease of their blood circulation time and significant reduction of liver accumulation [220]. DeSimone et al. carried out a comprehensive study on the influence of PEG density on protein binding, RES uptake, pharmacokinetics, and the biodistribution of nanocarriers prepared by PRINT technology [221]. Their findings indicated that, unlike NPs with lower PEG density, the counterparts with greater PEG density demonstrated lower absorption levels of albumin proteins, leading to greater blood circulation time and lower liver accumulation. Since the length and density of PEG has a potent effect on NMs’ half-life and biodistribution, it has been reported that these properties also make a substantial impact on the targeting and accumulation of NPs in the affected areas, including tumors [222, 223]. It’s worth noting, however, that a PEG with a density that is too high may not be appropriate for lengthening circulation or increasing accumulation in desired sites. High PEG density may result in the imbalance between the hydrophilicity and hydrophobicity of NMs, leading to physicochemical instability and aggregation during circulation, leading to a rapid elimination by RES [224, 225].

### Drug release-dependent pharmaceutical evaluation of NMs under the bioimaging guidance

Usually, the drug-release pattern is investigated by pharmacokinetic studies that measure time-dependent plasma drug concentrations by monitoring the drug molecules themselves [226, 227]. The pharmacokinetic results, however, can vary significantly from the local ones if the drug molecules are expected to be enriched or depleted at certain local sites. This is of great importance in the treatment of cancer since the local drug concentration at the tumor tissue directly impacts therapeutic efficacy. Thus, successful chemotherapy remains challenging due to unknown drug concentrations at the tumor site, which may be insufficient or excessive. The commonly used approaches to estimate the drug release and monitor the dynamics of changes in drug concentration are extremely difficult. Generally, harvested tissues must be homogenized and cells must be lysed to release the drug from specific intracellular compartments. Throughout these processes, especially in the process of cell lysis, the intact drug carriers will also be destroyed, causing great difficulties in differentiating the released drug agents from the inactive compounds remaining in the carriers during tissue collection [228–230]. In this regard, the development of non-invasive techniques to monitor the drug release and determine its concentration kinetics in localized tissues is of crucial importance.

**Fig. 13:**
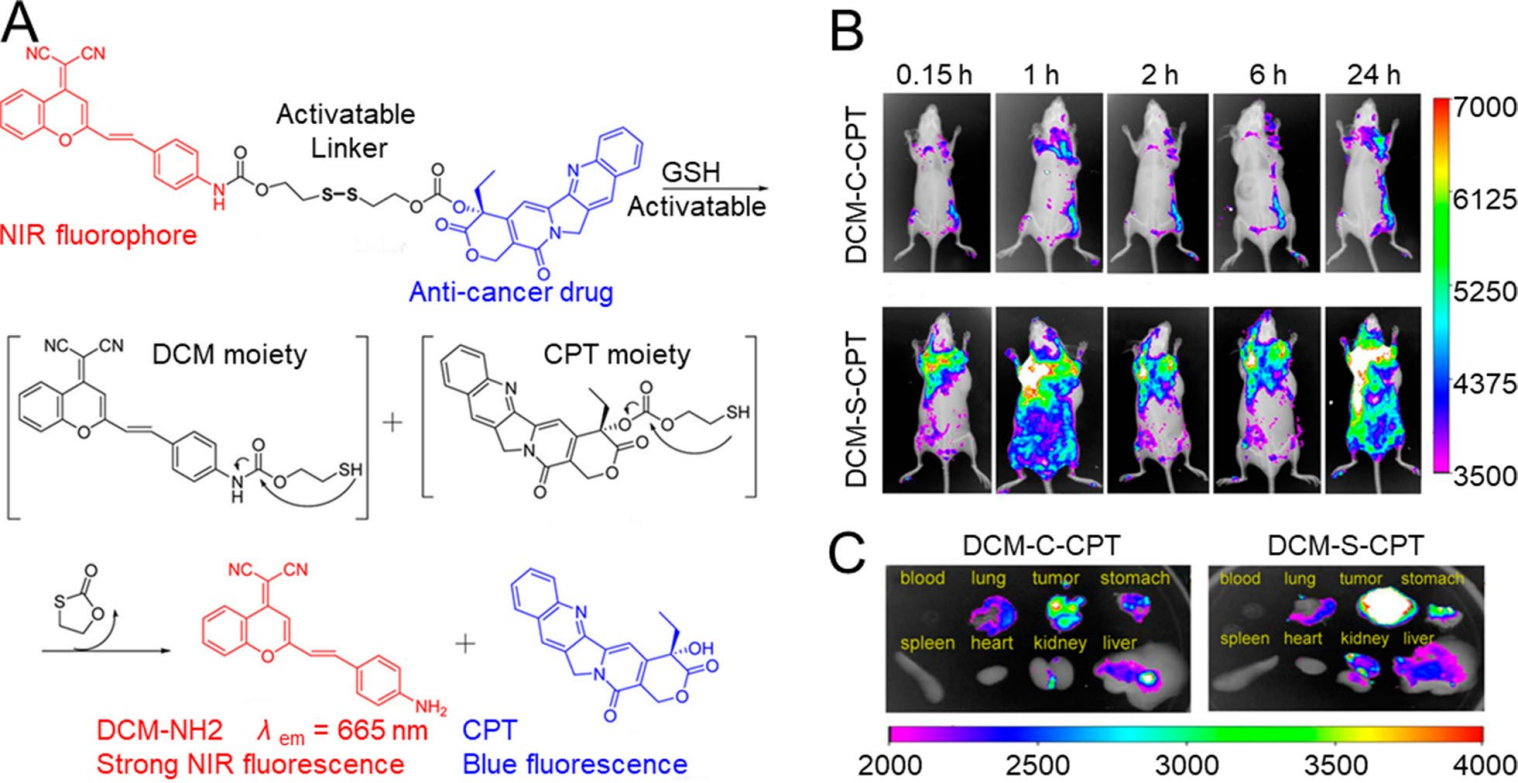
Drug release-dependent biodistribution of NMs. **A** release mechanism of CPT from activatable prodrug by treatment with GSH. In vivo imaging of tumor-bearing mice at different times (0.15, 1, 2, 6, and 24 h) after i.v. injection of: **B** DCM-C-CPT (0.08 mg/ kg) and DCM-S-CPT (0.08 mg/kg). **C** fluorescence images of the internal organs after anatomy for DCM-C-CPT and DCM-S-CPT. Reproduced with permission from [30]. Copyright 2014, American Chemical Society

Tracking drug concentration in real time is critical to adjusting the in vivo drug-release rate and optimal concentration for treatment. NIR fluorescence imaging techniques, including the PA method, can provide real-time tracking of drug delivery by taking advantage of the deep tissue penetration, high contrast, and decreased background auto-fluorescence interference [231]. The monitoring of drug concentration by directly tracking drug molecules with radionuclides or fluorochromes prior to entrapment by NMs might be a promising strategy for semi-quantitatively detecting active drug release in a non-invasive manner. By linking latent fluorophores with the drug molecules through the activatable bond, real-time information regarding the drug-release procedure can be received by using non-invasive fluorescence-detection modalities. NIR fluorophores are often exploited as fluorescent probes for drug-release monitoring in biological systems since NIR photons can penetrate deeply into the skin and underlying tissues, causing minimum damage to the biological samples while possessing low background interference. For instance, Wu et al. conjugated a NIR fluorophore – dicyanometh-ylene-4 H-pyran derivative (DCM) – with anti-cancer camptothecin (CPT) through a disulfide linker (DCM-S-CPT) and loaded this prodrug into polyethylene glycol-polylactic acid (PEG-PLA) NPs [30]. The drug-release profile and cancer therapeutic efficacy was analyzed *via* NIR fluorescence *in situ* and in vivo (Fig. 13).

Zhang and colleagues fabricated a real-time drug-reporting conjugate (CPT-SS-CyN), consisting of a model therapeutic drug CPT, a disulfide linker, and a NIR fluorescent cyanine-amine dye (CyN) as a diagnostic tool for quantitative CPT release analysis [232]. This disulfide bond between CPT and the NIR dye is sensitive to intracellular reducing agents (e.g., glutathione, cysteine, and thioredoxin) and can be cleaved under their treatment, followed by drug and dye release. A linear relationship between the amount of released CPT and fluorescence intensity at 760 nm was detected, which allows for the quantitative monitoring of CPT release cells and semi-quantitative analysis in live organisms in a non-invasive and real-time manner.

**Fig. 14:**
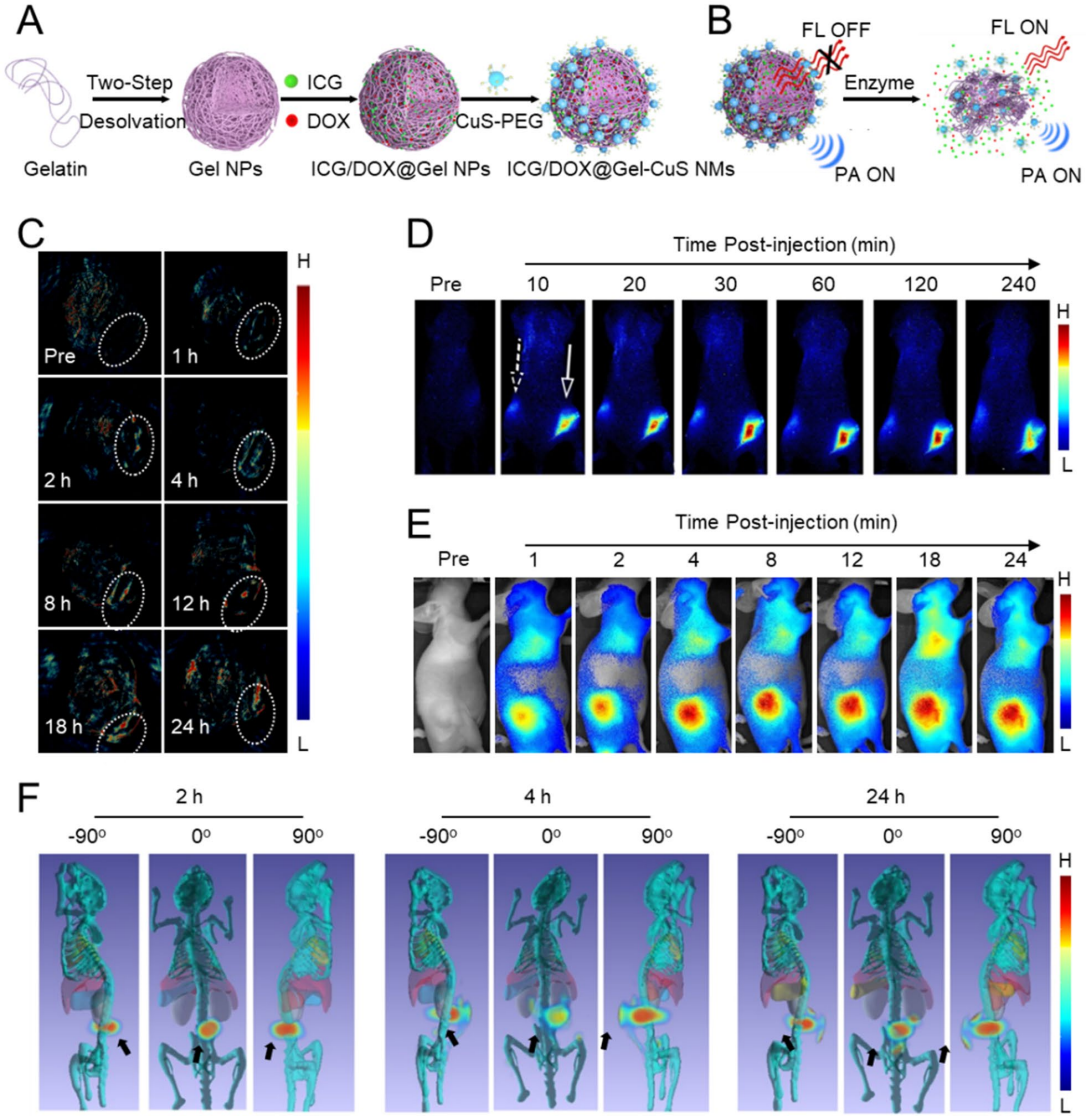
Drug release-dependent intratumoral accumulation of NMs. Schematic representation of: **A** ICG/DOX@Gel-CuS NMs fabrication, **B** mechanism of enzyme-activated DOX release followed by real-time monitoring and quantification by fluorescence/PA dual-modal imaging. **C** in vivo MSOT images of mice administered ICG/DOX@Gel-CuS NMs at various time points p.i. **D** in vivo NIR fluorescence images of mice administered NMs *via* both subcutaneous (dotted arrow) and intratumor (solid arrow) injection at various time points p.i. **E** NIR fluorescence images of mice administered ICG/DOX@ Gel-CuS NMs through i.v. injection at various time points p.i. **F** 3D-reconstruction of transillumination fluorescence images of mice administered NMs through i.v. injection at various tilt angles. Reproduced with permission from [233]. Copyright 2018, American Chemical Society

Another strategy to evaluate drug-release behaviors is to construct multiple stimuli-sensitive NMs. Drug release will be instantly reflected by responsive imaging signals that are functionally related to the drug’s existence (released or entrapped). External cues for triggering drug release (e.g., temperature, magnetic field, ultrasound, or light) may, however, create additional obstacles for clinical translation since the therapeutic testing and subsequent use on patients must be carried out exclusively in clinics using specialized equipment. In this regard, it would be highly applicable to provide real-time tracking of drug release in NMs in response to internal stimuli (e.g., acidic pH, redox species, or enzymes) exhibited only by the pathological tissue. Li et al. fabricated an enzyme-activatable core-satellite fluorescence/PA dual-modal imaging ICG/DOX@Gel-CuS NMs that is comprised of gelatin NPs loaded with NIR dye indocyanine green (ICG) and chemo drug DOX and decorated with “satellite” CuS NPs [233]. After injection into MDA-MB-231 tumor-bearing mice, the ICG/DOX@Gel-CuS accumulated in the tumor, where the overexpressed enzymes induced the degradation of the gelatin framework, followed by DOX and ICG release, which was observed with the ICG fluorescence signal at the tumor site (Fig. 14). Furthermore, Yan et al. formulated a dual-stimuli smart nanoprobe P(Cy-S-CPT) comprised of an ionizable tertiary amine-containing diblock copolymer and a dual-channel NIR fluorescence agent, Cy-S-CPT [234]. The controllable drug release was evaluated using the 3D imaging and in vivo fluorescence, and the results revealed a precise target tumor ability of P(Cy-S-CPT) as well as controllable drug-release behavior.

Recently, different NPs-based contrast probes have been developed to track drug-release kinetics applying MRI *via* interaction between MRI contrast species and drug molecules [235–237]. In particular, stimuli-responsive MRI nanosystems were fabricated in which MR signals were turned off by complexing or encapsulating with drugs to conceal the paramagnetic ions from the surrounding water molecules. Followed by exogenous or endogenous stimuli activation–including temperature, light, pH, and small molecules–the MR signals exhibit marked differences along with the release or exposure of paramagnetic ions. These features make MRI potentially applicable for real-time drug-release monitoring.

## Conclusion and perspectives

Imaging modalities have been successfully applied in preclinical studies on the biodistribution and pharmacokinetics of NMs, exhibiting their value for this purpose. Advancements in real-time in vivo visualization can enable extensive characterization of NMs’ behavior in biological systems. By applying state-of-the-art imaging techniques, it is possible to visualize properly labeled NMs over time throughout the body, which can allow for the quantification of their kinetic parameters, facilitate the assessment of transport and physiological properties, and provide greater insights into the intrinsic interplay between structural and functional relationships. This allows for the development and accurate construction of NMs with desired physicochemical properties that maximize their intended therapeutic benefits. Incorporating imaging techniques into the pharmaceutical evaluation of NMs will undoubtedly result in additional valuable information that conventional methods, such as blood sampling, cannot provide. This concept would significantly accelerate early-stage development and advance the path to successful clinical translation. Despite the remarkable potential of imaging-based pharmaceutical evaluation of NMs, there are issues that need to be addressed during the quantification and interpretation of imaging results.

Firstly, a general limitation of the imaging-guided estimations is the stability of the bond between the imaging probe and the NMs it labels. Bond stability is crucial for the accurate assessment of distribution kinetics and parameter values, and its breakage can lead to leakage of the imaging agent from the NMs, confounding observations and leading to inaccurate results. Furthermore, the imaging tag is most often attached to the nanocarrier rather than the drug, while the localization of the drug may differ from that of the nanocarrier after release. The biodistribution of NMs at early time points following their administration is of great importance for the accurate evaluation of pharmacokinetics. Thus, imaging techniques with low temporal resolution should be avoided in such circumstances and can only be used when a semi-quantitative evaluation is required.

Secondly, since each modality has specific advantages and limitations, the appropriate choice of imaging technique is intensely associated with the physicochemical properties of NMs, labeling methods, and spatial level (organ, tissue or cellular level). The image resolution and study duration should also be considered depending on the assessment objectives. For example, MRI has limited applications in monitoring the biodistribution of NMs due to its poor sensitivity and the difficulties of providing whole-body imaging. The low sensitivity of CT imaging results in the need for high doses of contrast agents, which can lead to potential contrast-agent-related toxicities and limitations for imaging-guided pharmaceutical evaluations of NMs. Compared with MRI and CT, the nuclear imaging techniques have the best sensitivity and allow for the visible pharmaceutic evaluation of the NMs at trace-amount. However, nuclear imaging suffers from low spatial resolution, lack of anatomical information, and the necessity of using radioactive agents. The former two can be overcome by combining PET/SPECT with CT or MRI to provide more detailed information about the anatomical and spatial distribution of the NMs. The fundamental limitations of the use of FLI for preclinical evaluations lie on the poor penetration depth of excitation/emission lights, tissue autofluorescence, and diffusive scattering of fluorescence signals in the body. These disadvantages result in inability of FLI to correctly assign the probe accumulation to specific deeper-seated tissues and organs, as well as to perform an absolute quantification of probe biodistribution and accumulation.

Thirdly, a formidable challenge is controlling and keeping constant key physicochemical properties of NMs. Since the particle size distribution, shape, and surface features have an enormous influence on targeting properties, drug release rate, biodistribution, and excretion of NMs, it is of great importance to maintain the uniformity of these characteristics to perform accurate pharmaceutical assessments.

Finally, it is important to keep in mind that biomedical imaging modalities are mainly focused on monitoring pharmacokinetic and pharmacodynamic behavior of NMs and they have a potential to be integrated in preclinical drug delivery research to improve NMs-based treatments. However, non-invasive imaging techniques cannot provide an adequate assessment of NMs toxicity due to complex evaluation procedures that are beyond the functionality of imaging methods. At present, classical drug toxicity assays, such as histopathology examination, are used to evaluate NMs toxicological profiles.

## Data Availability

Not applicable.

## Abbreviations

NMs: Nanomedicines
FDA: Food and Drug Administration
PA: Photoacoustic
MPS: Mononuclear phagocyte system
NPs: Nanoparticles
MRI: Magnetic resonance imaging
MIONs: Monocrystalline iron oxide NPs
SPIOs: Superparamagnetic iron oxides
USPIOs: Ultrasmall superparamagnetic iron oxides
FLI: Fluorescence imaging
NIR: Near-infrared
HOMO: Highest occupied molecular orbital
LUMO: Lowest unoccupied molecular orbital
PET: Positron emission tomography
SPECT: Single photon emission computed tomography
DTPA: Diethylenetriaminepentaacetic acid
CT: Computed tomography
NMR: Nuclear magnetic resonance
SWNT: Semiconducting single-walled carbon nanotubes
EPR: Enhanced permeability and retention
CLSM: Confocal laser scanning microscopy
MSOT: Multispectral optical tomography
QDs: Quantum dots
TEM: Transmission electron microscopy
mNRs: Multiple nanorods
BiS: Bismuth sulfide
HSA: Human serum albumin
DTX: Docetaxel
DA: Diazirine
TPZ: Tirapazamine
MSNs: Mesoporous silica nanoparticles
CHT-EC: Caudal hematopoietic tissue endothelial cells
DLAV: Dorsal longitudinal anastomotic vessel
ISV: Intersegmental vessel
CV: Caudal vein
PLGA: Poly(lactic-co-glycolic acid)
NAcHis-TPGS: N-acetyl histidine modified D-α-tocopheryl polyethylene glycol succinate
NIRFs: Near-infrared fluorophores
PNCs: Protein nanocages
CPT: Camptothecin
CyN: Cyanine-amine dye
ICG: Indocyanine green

## References

[1] Mitchell MJ, Billingsley MM, Haley RM, Wechsler ME, Peppas NA, Langer R (2021). Engineering precision nanoparticles for drug delivery. Nat Rev Drug Discovery.

[2] Manzari MT, Shamay Y, Kiguchi H, Rosen N, Scaltriti M, Heller DA (2021). Targeted drug delivery strategies for precision medicines. Nat Rev Mater.

[3] Deng C, Zhang Q, He P, Zhou B, He K, Sun X (2021). Targeted apoptosis of macrophages and osteoclasts in arthritic joints is effective against advanced inflammatory arthritis. Nat Commun.

[4] Wang Z, Little N, Chen J, Lambesis KT, Le KT, Han W (2021). Immunogenic camptothesome nanovesicles comprising sphingomyelin-derived camptothecin bilayers for safe and synergistic cancer immunochemotherapy. Nat Nanotechnol.

[5] Zhou Z, Yeh C-F, Mellas M, Oh M-J, Zhu J, Li J (2021). Targeted polyelectrolyte complex micelles treat vascular complications in vivo. Proceed Nat Academy Sci..

[6] Rabinow BE (2004). Nanosuspensions in drug delivery. Nat Rev Drug Discovery.

[7] Soares S, Sousa J, Pais A, Vitorino C (2018). Nanomedicine: principles, properties, and regulatory issues. Front Chem.

[8] Nel AE, Mädler L, Velegol D, Xia T, Hoek EM, Somasundaran P (2009). Understanding biophysicochemical interactions at the nano–bio interface. Nat Mater.

[9] Poon W, Kingston BR, Ouyang B, Ngo W, Chan WCW (2020). A framework for designing delivery systems. Nat Nanotechnol..

[10] Myerson JW, Patel PN, Rubey KM, Zamora ME, Zaleski MH, Habibi N (2022). Supramolecular arrangement of protein in nanoparticle structures predicts nanoparticle tropism for neutrophils in acute lung inflammation. Nat Nanotechnol.

[11] Shi J, Kantoff PW, Wooster R, Farokhzad OC (2017). Cancer nanomedicine: progress, challenges and opportunities. Nat Rev Cancer.

[12] Bobo D, Robinson KJ, Islam J, Thurecht KJ, Corrie SR (2016). Nanoparticle-based medicines: a review of FDA-approved materials and clinical trials to date. Pharmaceut Res.

[13] Li S, Chen T, Wang Y, Liu L, Lv F, Li Z (2017). Conjugated polymer with intrinsic alkyne units for synergistically enhanced Raman imaging in living cells. Angew Chem Int Edit.

[14] Zhang Y, Wang X, Chu C, Zhou Z, Chen B, Pang X (2020). Genetically engineered magnetic nanocages for cancer magneto-catalytic theranostics. Nat Commun.

[15] Marcos-Contreras OA, Greineder CF, Kiseleva RY, Parhiz H, Walsh LR, Zuluaga-Ramirez V (2020). Selective targeting of nanomedicine to inflamed cerebral vasculature to enhance the blood–brain barrier. Proceed Nat Academy Sci..

[16] Ren H, Zeng X-Z, Zhao X-X, Hou D-y, Yao H, Yaseen M (2022). A bioactivated in vivo assembly nanotechnology fabricated NIR probe for small pancreatic tumor intraoperative imaging. Nat Commun.

[17] Song Z, Liu T, Lai H, Meng X, Yang L, Su J (2022). A Universally EDTA-assisted synthesis of polytypic bismuth telluride nanoplates with a size-dependent enhancement of tumor radiosensitivity and metabolism in vivo. ACS nano..

[18] Barenholz YC. Doxil®—the first FDA-approved nano-drug: from an idea to a product. Handbook of harnessing biomaterials in nanomedicine. Jenny Stanford Publishing; 2021. pp. 463–528.

[19] Marques MR, Choo Q, Ashtikar M, Rocha TC, Bremer-Hoffmann S, Wacker MG (2019). Nanomedicines-tiny particles and big challenges. Adv Drug Deliver Rev.

[20] Anselmo AC, Mitragotri S (2021). Nanoparticles in the clinic: An update post COVID-19 vaccines. Bioeng translational Med.

[21] D’Mello SR, Cruz CN, Chen M-L, Kapoor M, Lee SL, Tyner KM (2017). The evolving landscape of drug products containing nanomaterials in the United States. Nat Nanotechnol.

[22] Germain M, Caputo F, Metcalfe S, Tosi G, Spring K, Åslund AK (2020). Delivering the power of nanomedicine to patients today. J Control Release.

[23] Ioannidis JPA, Kim BYS, Trounson A (2018). How to design preclinical studies in nanomedicine and cell therapy to maximize the prospects of clinical translation. Nat Biomedical Eng.

[24] Wu L-P, Wang D, Li Z (2020). Grand challenges in nanomedicine. Mater Sci Engineering: C.

[25] Treuel L, Eslahian K, Docter D, Lang T, Zellner R, Nienhaus K (2014). Physicochemical characterization of nanoparticles and their behavior in the biological environment. Phys Chem Chem Phys.

[26] Hua S, De Matos MB, Metselaar JM, Storm G (2018). Current trends and challenges in the clinical translation of nanoparticulate nanomedicines: pathways for translational development and commercialization. Front Pharmacol.

[27] Coty J-B, Vauthier C (2018). Characterization of nanomedicines: A reflection on a field under construction needed for clinical translation success. J Control Release.

[28] Kunjachan S, Ehling J, Storm G, Kiessling F, Lammers T (2015). Noninvasive imaging of nanomedicines and nanotheranostics: principles, progress, and prospects. Chem Rev.

[29] Zhang T, Wang Z, Xiang H, Xu X, Zou J, Lu C (2021). Biocompatible superparamagnetic europium-doped iron oxide nanoparticle clusters as multifunctional nanoprobes for multimodal in vivo imaging. ACS Appl Mater Inter.

[30] Wu X, Sun X, Guo Z, Tang J, Shen Y, James TD (2014). In vivo and in situ tracking cancer chemotherapy by highly photostable NIR fluorescent theranostic prodrug. J Am Chem Soc.

[31] Seo JW, Mahakian LM, Tam S, Qin S, Ingham ES, Meares CF (2015). The pharmacokinetics of Zr-89 labeled liposomes over extended periods in a murine tumor model. Nucl Med Biol.

[32] Zhu W, Yang Y, Jin Q, Chao Y, Tian L, Liu J (2019). Two-dimensional metal-organic-framework as a unique theranostic nano-platform for nuclear imaging and chemo-photodynamic cancer therapy. Nano Res.

[33] Gao Y, Kang J, Lei Z, Li Y, Mei X, Wang G (2020). Use of the highly biocompatible Au nanocages@ PEG nanoparticles as a new contrast agent for in vivo computed tomography scan imaging. Nanoscale Res Lett.

[34] Yim G, Kang S, Kim Y-J, Kim Y-K, Min D-H, Jang H (2019). Hydrothermal Galvanic-Replacement-Tethered Synthesis of Ir–Ag–IrO2 Nanoplates for Computed Tomography-Guided Multiwavelength Potent Thermodynamic Cancer Therapy. ACS Nano.

[35] Van Geuns R-JM, Wielopolski PA, de Bruin HG, Rensing BJ, van Ooijen PM, Hulshoff M (1999). Basic principles of magnetic resonance imaging. Prog Cardiovasc Dis.

[36] Wang Z, Xue X, Lu H, He Y, Lu Z, Chen Z (2020). Two-way magnetic resonance tuning and enhanced subtraction imaging for non-invasive and quantitative biological imaging. Nat Nanotechnol..

[37] Kiru L, Zlitni A, Tousley AM, Dalton GN, Wu W, Lafortune F (2022). In vivo imaging of nanoparticle-labeled CAR T cells. Proceed Nat Acad Sci..

[38] Terreno E, Castelli DD, Viale A, Aime S (2010). Challenges for molecular magnetic resonance imaging. Chem Rev.

[39] Wei H, Wiśniowska A, Fan J, Harvey P, Li Y, Wu V (2021). Single-nanometer iron oxide nanoparticles as tissue-permeable MRI contrast agents. Proceed Nat Acad Sci..

[40] Wang J, Jia Y, Wang Q, Liang Z, Han G, Wang Z (2021). An Ultrahigh-Field‐Tailored T1–T2 Dual‐Mode MRI Contrast Agent for High‐Performance Vascular Imaging. Adv Mater.

[41] Shin T-H, Choi Y, Kim S, Cheon J (2015). Recent advances in magnetic nanoparticle-based multi-modal imaging. Chem Soc Rev.

[42] Kiessling F, Mertens ME, Grimm J, Lammers T (2014). Nanoparticles for imaging: top or flop?. Radiology.

[43] Lux J, Sherry AD (2018). Advances in gadolinium-based MRI contrast agent designs for monitoring biological processes in vivo. Curr Opin Chem Biol.

[44] Lee SH, Kim BH, Na HB, Hyeon T (2014). Paramagnetic inorganic nanoparticles as T1 MRI contrast agents. Wiley Interdisciplinary Reviews: Nanomedicine and Nanobiotechnology.

[45] Marasini S, Yue H, Ghazanfari A, Ho SL, Park J, Kim S (2021). Polyaspartic Acid-Coated Paramagnetic Gadolinium Oxide Nanoparticles as a Dual-Modal T1 and T2 Magnetic Resonance Imaging Contrast Agent. Appl Sci.

[46] Park JY, Baek MJ, Choi ES, Woo S, Kim JH, Kim TJ (2009). Paramagnetic ultrasmall gadolinium oxide nanoparticles as advanced T 1 MRI contrast agent: account for large longitudinal relaxivity, optimal particle diameter, and in vivo T 1 MR images. ACS Nano.

[47] Yang J, Shan P, Zhao Q, Zhang S, Li L, Yang X (2021). A design strategy of ultrasmall Gd2O3 nanoparticles for T1 MRI with high performance. New J Chem.

[48] Zhang H, Zhang J, Chen Y, Wu T, Lu M, Chen Z, et al. Hollow Carbon Nanospheres Embedded with Stoichiometric γ-Fe2O3 and GdPO4: Tuning the Nanosphere for In-vitro and In-vivo Size Effect Evaluation. Nanoscale Adv. 2022. 10.1039/d1na00771h PMC9417868 36133683

[49] Cheung ENM, Alvares RD, Oakden W, Chaudhary R, Hill ML, Pichaandi J (2010). Polymer-stabilized lanthanide fluoride nanoparticle aggregates as contrast agents for magnetic resonance imaging and computed tomography. Chem Mater.

[50] Cai X, Zhu Q, Zeng Y, Zeng Q, Chen X, Zhan Y (2019). Manganese oxide nanoparticles as MRI contrast agents in tumor multimodal imaging and therapy. Int J Nanomed.

[51] Mauri M, Collico V, Morelli L, Das P, Garcia I, Penaranda Avila J (2020). MnO Nanoparticles Embedded in Functional Polymers as T 1 Contrast Agents for Magnetic Resonance Imaging. ACS Appl Nano Mater.

[52] Wei R, Liu K, Zhang K, Fan Y, Lin H, Gao J (2022). Zwitterion-Coated Ultrasmall MnO nanoparticles enable highly sensitive T 1-weighted contrast-enhanced brain imaging. ACS Appl Mater Inter..

[53] Jain P, Patel K, Jangid AK, Guleria A, Patel S, Pooja D (2020). Modulating the delivery of 5-fluorouracil to human colon cancer cells using multifunctional arginine-coated manganese oxide nanocuboids with MRI properties. ACS Appl Bio Mater.

[54] Xiao J, Tian X, Yang C, Liu P, Luo N, Liang Y (2013). Ultrahigh relaxivity and safe probes of manganese oxide nanoparticles for in vivo imaging. Sci Rep.

[55] Ji S, Chen Y, Zhao X, Cai Y, Zhang X, Sun F (2021). Surface morphology and payload synergistically caused an enhancement of the longitudinal relaxivity of a Mn 3 O 4/PtO x nanocomposite for magnetic resonance tumor imaging. Biomaterials Sci.

[56] Vargo KB, Zaki AA, Warden-Rothman R, Tsourkas A, Hammer DA (2015). Superparamagnetic iron oxide nanoparticle micelles stabilized by recombinant oleosin for targeted magnetic resonance imaging. Small.

[57] Smith BR, Gambhir SS (2017). Nanomaterials for in vivo imaging. Chem Rev.

[58] Smith BR, Heverhagen J, Knopp M, Schmalbrock P, Shapiro J, Shiomi M (2007). Localization to atherosclerotic plaque and biodistribution of biochemically derivatized superparamagnetic iron oxide nanoparticles (SPIONs) contrast particles for magnetic resonance imaging (MRI). Biomed Microdevices.

[59] Ahrens ET, Bulte JW (2013). Tracking immune cells in vivo using magnetic resonance imaging. Nat Rev Immunol.

[60] Daldrup-Link HE, Golovko D, Ruffell B, DeNardo DG, Castaneda R, Ansari C (2011). MRI of tumor-associated macrophages with clinically applicable iron oxide nanoparticles. Clin Cancer Res.

[61] Kim SJ, Lewis B, Steiner MS, Bissa UV, Dose C, Frank JA (2016). Superparamagnetic iron oxide nanoparticles for direct labeling of stem cells and in vivo MRI tracking. Contrast Media Mol Imaging.

[62] Hao X, Xu B, Chen H, Wang X, Zhang J, Guo R (2019). Stem cell-mediated delivery of nanogels loaded with ultrasmall iron oxide nanoparticles for enhanced tumor MR imaging. Nanoscale.

[63] Song G, Kenney M, Chen Y-S, Zheng X, Deng Y, Chen Z (2020). Carbon-coated FeCo nanoparticles as sensitive magnetic-particle-imaging tracers with photothermal and magnetothermal properties. Nat biomedical Eng.

[64] Piché D, Tavernaro I, Fleddermann J, Lozano JG, Varambhia A, Maguire ML (2019). Targeted T 1 Magnetic Resonance Imaging Contrast Enhancement with Extraordinarily Small CoFe2O4 Nanoparticles. ACS Appl Mater Inter.

[65] Dan S, Naskar J, Kamsonlian S, Chattree A. Comparative study of ferromagnetic behaviour in bare and PMMA modified manganese ferrite (MnFe2O4) nanoparticles. Int Nano Lett. 2021:1–11.

[66] Sitthichai S, Junploy P, Thongtem T, Pilapong C, Phuruangrat A, Thongtem S. Synthesis and Characterization of NiFe2O4 Magnetic Nanoparticles for Magnetic Resonance Imaging Application. Int J Nanosci. 2021:2150047.

[67] Slabu I, Wiemer K, Steitz J, Liffmann R, Mues B, Eisold S (2019). Size-tailored biocompatible FePt nanoparticles for dual T 1/T 2 magnetic resonance imaging contrast enhancement. Langmuir.

[68] Shin T-H, Kim PK, Kang S, Cheong J, Kim S, Lim Y (2021). High-resolution T1 MRI via renally clearable dextran nanoparticles with an iron oxide shell. Nat Biomed Engineer..

[69] Kim JH, Dodd S, Ye FQ, Knutsen AK, Nguyen D, Wu H (2021). Sensitive detection of extremely small iron oxide nanoparticles in living mice using MP2RAGE with advanced image co-registration. Scientific Reports..

[70] Meng F, Wang J, Ping Q, Yeo Y (2018). Quantitative assessment of nanoparticle biodistribution by fluorescence imaging, revisited. ACS Nano.

[71] Gao X, Cui R, Ji G, Liu Z (2018). Size and surface controllable metal–organic frameworks (MOFs) for fluorescence imaging and cancer therapy. Nanoscale.

[72] Park S-m, Aalipour A, Vermesh O, Yu JH, Gambhir SS (2017). Towards clinically translatable in vivo nanodiagnostics. Nat Reviews Mater.

[73] Wang S, Liu L, Fan Y, El-Toni AM, Alhoshan MS, Li D (2019). In vivo high-resolution ratiometric fluorescence imaging of inflammation using NIR-II nanoprobes with 1550 nm emission. Nano Lett.

[74] Diao S, Blackburn JL, Hong G, Antaris AL, Chang J, Wu JZ (2015). Fluorescence imaging in vivo at wavelengths beyond 1500 nm. Angew Chem.

[75] Peng H-S, Chiu DT (2015). Soft fluorescent nanomaterials for biological and biomedical imaging. Chem Soc Rev.

[76] Chen G, Roy I, Yang C, Prasad PN (2016). Nanochemistry and nanomedicine for nanoparticle-based diagnostics and therapy. Chem Rev.

[77] Thimsen E, Sadtler B, Berezin MY (2017). Shortwave-infrared (SWIR) emitters for biological imaging: a review of challenges and opportunities. Nanophotonics.

[78] Huang J, Jiang Y, Li J, Huang J, Pu K (2021). Molecular Chemiluminescent Probes with a Very Long Near-Infrared Emission Wavelength for in Vivo Imaging. Angew Chem Int Edit.

[79] Ogawa M, Kosaka N, Choyke PL, Kobayashi H (2009). In vivo molecular imaging of cancer with a quenching near-infrared fluorescent probe using conjugates of monoclonal antibodies and indocyanine green. Cancer Res.

[80] Ishizawa T, Fukushima N, Shibahara J, Masuda K, Tamura S, Aoki T (2009). Real-time identification of liver cancers by using indocyanine green fluorescent imaging. Cancer.

[81] An F, Yang Z, Zheng M, Mei T, Deng G, Guo P (2020). Rationally assembled albumin/indocyanine green nanocomplex for enhanced tumor imaging to guide photothermal therapy. J Nanobiotechnol.

[82] Xiao YF, An FF, Chen JX, Yu J, Tao WW, Yu Z (2019). The Nanoassembly of an Intrinsically Cytotoxic Near-Infrared Dye for Multifunctionally Synergistic Theranostics. Small.

[83] Li B, Lu L, Zhao M, Lei Z, Zhang F (2018). An Efficient 1064 nm NIR-II Excitation Fluorescent Molecular Dye for Deep-Tissue High-Resolution Dynamic Bioimaging. Angewandte Chemie..

[84] Liu S, Ou H, Li Y, Zhang H, Liu J, Lu X (2020). Planar and twisted molecular structure leads to the high brightness of semiconducting polymer nanoparticles for NIR-IIa fluorescence imaging. J Am Chem Soc.

[85] Antaris AL, Chen H, Diao S, Ma Z, Zhang Z, Zhu S (2017). A high quantum yield molecule-protein complex fluorophore for near-infrared II imaging. Nat Commun.

[86] Fan Y, Wang P, Lu Y, Wang R, Zhou L, Zheng X (2018). Lifetime-engineered NIR-II nanoparticles unlock multiplexed in vivo imaging. Nat Nanotechnol.

[87] Huang J, Lyu Y, Li J, Cheng P, Jiang Y, Pu K (2019). A Renal-Clearable Duplex Optical Reporter for Real‐Time Imaging of Contrast‐Induced Acute Kidney Injury. Angew Chem.

[88] Zhu S, Tian R, Antaris AL, Chen X, Dai H (2019). Near-infrared‐II molecular dyes for cancer imaging and surgery. Adv Mater.

[89] Welsher K, Liu Z, Daranciang D, Dai H (2008). Selective probing and imaging of cells with single walled carbon nanotubes as near-infrared fluorescent molecules. Nano Lett.

[90] Tan J, Li Q, Meng S, Li Y, Yang J, Ye Y (2021). Time-dependent phosphorescence colors from carbon dots for advanced dynamic information encryption. Adv Mater.

[91] Dai X, Zhao X, Liu Y, Chen B, Ding X, Zhao N (2021). Controlled Synthesis and Surface Engineering of Janus Chitosan-Gold Nanoparticles for Photoacoustic Imaging‐Guided Synergistic Gene/Photothermal Therapy. Small.

[92] Levy ES, Tajon CA, Bischof TS, Iafrati J, Fernandez-Bravo A, Garfield DJ (2016). Energy-looping nanoparticles: harnessing excited-state absorption for deep-tissue imaging. ACS Nano.

[93] Tao Z, Dang X, Huang X, Muzumdar MD, Xu ES, Bardhan NM (2017). Early tumor detection afforded by in vivo imaging of near-infrared II fluorescence. Biomaterials.

[94] Li Q, Li X, Zhang L, Zuo J, Zhang Y, Liu X (2018). An 800 nm driven NaErF 4@ NaLuF 4 upconversion platform for multimodality imaging and photodynamic therapy. Nanoscale.

[95] Zhou J, Jiang Y, Hou S, Upputuri PK, Wu D, Li J (2018). Compact plasmonic blackbody for cancer theranosis in the near-infrared II window. ACS Nano.

[96] Mu CJ, LaVan DA, Langer RS, Zetter BR (2010). Self-assembled gold nanoparticle molecular probes for detecting proteolytic activity in vivo. ACS Nano.

[97] Zheng Z, Jia Z, Qu C, Dai R, Qin Y, Rong S (2021). Biodegradable Silica-Based Nanotheranostics for Precise MRI/NIR‐II Fluorescence Imaging and Self‐Reinforcing Antitumor Therapy. Small.

[98] Santos HD, Zabala Gutiérrez I, Shen Y, Lifante J, Ximendes E, Laurenti M (2020). Ultrafast photochemistry produces superbright short-wave infrared dots for low-dose in vivo imaging. Nat Commun.

[99] Hong G, Antaris AL, Dai H (2017). Near-infrared fluorophores for biomedical imaging. Nat biomedical Eng.

[100] Yang Q, Hu Z, Zhu S, Ma R, Ma H, Ma Z (2018). Donor engineering for NIR-II molecular fluorophores with enhanced fluorescent performance. J Am Chem Soc.

[101] Antaris AL, Chen H, Cheng K, Sun Y, Hong G, Qu C (2016). A small-molecule dye for NIR-II imaging. Nat Mater.

[102] Zhu S, Yang Q, Antaris AL, Yue J, Ma Z, Wang H (2017). Molecular imaging of biological systems with a clickable dye in the broad 800-to 1,700-nm near-infrared window. Proceed Nat Acad Sci.

[103] Qian G, Dai B, Luo M, Yu D, Zhan J, Zhang Z (2008). Band gap tunable, donor – acceptor – donor charge-transfer heteroquinoid-based chromophores: near infrared photoluminescence and electroluminescence. Chem Mater.

[104] Michaeli K, Beratan DN, Waldeck DH, Naaman R (2019). Voltage-induced long-range coherent electron transfer through organic molecules. Proceed Nat Acad Sci..

[105] Woo S-J, Park S, Jeong J-E, Hong Y, Ku M, Kim BY (2016). Synthesis and characterization of water-soluble conjugated oligoelectrolytes for near-infrared fluorescence biological imaging. ACS Appl Mater Inter.

[106] Sun Y, Ding M, Zeng X, Xiao Y, Wu H, Zhou H (2017). Novel bright-emission small-molecule NIR-II fluorophores for in vivo tumor imaging and image-guided surgery. Chem Sci.

[107] Sun Y, Qu C, Chen H, He M, Tang C, Shou K (2016). Novel benzo-bis (1, 2, 5-thiadiazole) fluorophores for in vivo NIR-II imaging of cancer. Chem Sci.

[108] He K, Chen S, Chen Y, Li J, Sun P, Lu X (2021). Water-Soluble Donor–Acceptor–Donor-Based Fluorophore for High-Resolution NIR-II Fluorescence Imaging Applications. ACS Appl Polym Mater.

[109] Zhou H, Yi W, Li A, Wang B, Ding Q, Xue L (2020). Specific Small-Molecule NIR‐II Fluorescence Imaging of Osteosarcoma and Lung Metastasis. Adv Healthc Mater.

[110] Pimlott SL, Sutherland A (2011). Molecular tracers for the PET and SPECT imaging of disease. Chem Soc Rev.

[111] Ge J, Zhang Q, Zeng J, Gu Z, Gao M (2020). Radiolabeling nanomaterials for multimodality imaging: New insights into nuclear medicine and cancer diagnosis. Biomaterials.

[112] Wang H, Kumar R, Nagesha D, Duclos RI, Sridhar S, Gatley SJ (2015). Integrity of (111)In-radiolabeled superparamagnetic iron oxide nanoparticles in the mouse. Nuclear Med biology.

[113] Black KC, Akers WJ, Sudlow G, Xu B, Laforest R, Achilefu S (2015). Dual-radiolabeled nanoparticle SPECT probes for bioimaging. Nanoscale.

[114] Wang JT-W, Rubio N, Kafa H, Venturelli E, Fabbro C, Ménard-Moyon C (2016). Kinetics of functionalised carbon nanotube distribution in mouse brain after systemic injection: Spatial to ultra-structural analyses. J Control Release.

[115] Dogra P, Adolphi NL, Wang Z, Lin Y-S, Butler KS, Durfee PN (2018). Establishing the effects of mesoporous silica nanoparticle properties on in vivo disposition using imaging-based pharmacokinetics. Nat Commun.

[116] Lacerda L, Soundararajan A, Singh R, Pastorin G, Al-Jamal KT, Turton J (2008). Dynamic imaging of functionalized multi‐walled carbon nanotube systemic circulation and urinary excretion. Adv Mater.

[117] Helbok A, Rangger C, von Guggenberg E, Saba-Lepek M, Radolf T, Thurner G (2012). Targeting properties of peptide-modified radiolabeled liposomal nanoparticles. Nanomed Nanotechnol Biol Med.

[118] Gill MR, Menon JU, Jarman PJ, Owen J, Skaripa-Koukelli I, Able S (2018). 111 In-labelled polymeric nanoparticles incorporating a ruthenium-based radiosensitizer for EGFR-targeted combination therapy in oesophageal cancer cells. Nanoscale.

[119] Ruan Q, Feng J, Jiang Y, Zhang X, Duan X, Wang Q (2021). Preparation and Bioevaluation of 99mTc-Labeled FAP Inhibitors as Tumor Radiotracers to Target the Fibroblast Activation Protein. Mol Pharm..

[120] Gao H, Liu X, Tang W, Niu D, Zhou B, Zhang H (2016). 99m Tc-conjugated manganese-based mesoporous silica nanoparticles for SPECT, pH-responsive MRI and anti-cancer drug delivery. Nanoscale.

[121] Guo Z, Chen M, Peng C, Mo S, Shi C, Fu G (2018). pH-sensitive radiolabeled and superfluorinated ultra-small palladium nanosheet as a high-performance multimodal platform for tumor theranostics. Biomaterials.

[122] Wang X, Jaraquemada-Peláez MadG, Rodríguez-Rodríguez C, Cao Y, Buchwalder C, Choudhary N (2018). H4octox: Versatile bimodal octadentate acyclic chelating ligand for medicinal inorganic chemistry. J Am Chem Soc.

[123] Gao F, Cai P, Yang W, Xue J, Gao L, Liu R (2015). Ultrasmall [64Cu] Cu nanoclusters for targeting orthotopic lung tumors using accurate positron emission tomography imaging. ACS Nano.

[124] Cao J, Wei Y, Zhang Y, Wang G, Ji X, Zhong Z (2019). Iodine-rich polymersomes enable versatile SPECT/CT imaging and potent radioisotope therapy for tumor in vivo. ACS Appl Mater Inter.

[125] Wang P, Sun W, Wang Q, Ma J, Su X, Jiang Q (2019). Iodine-labeled Au nanorods with high radiochemical stability for imaging-guided radiotherapy and photothermal therapy. ACS Appl Nano Mater.

[126] Mishiro K, Nishii R, Sawazaki I, Sofuku T, Fuchigami T, Sudo H (2022). Development of Radiohalogenated Osimertinib Derivatives as Imaging Probes for Companion Diagnostics of Osimertinib. J Med Chem..

[127] Simón M, Jørgensen JT, Norregaard K, Kjaer A (2020). 18F-FDG positron emission tomography and diffusion-weighted magnetic resonance imaging for response evaluation of nanoparticle-mediated photothermal therapy. Sci Rep.

[128] Chakravarty R, Chakraborty S, Ningthoujam RS, Vimalnath Nair K, Sharma KS, Ballal A (2016). Industrial-scale synthesis of intrinsically radiolabeled 64CuS nanoparticles for use in positron emission tomography (PET) imaging of cancer. Ind Eng Chem Res.

[129] Zhao Y, Sultan D, Detering L, Luehmann H, Liu Y (2014). Facile synthesis, pharmacokinetic and systemic clearance evaluation, and positron emission tomography cancer imaging of 64 Cu–Au alloy nanoclusters. Nanoscale.

[130] Suarez-Garcia S, Esposito TV, Neufeld-Peters J, Bergamo M, Yang H, Saatchi K (2021). Hybrid Metal–Phenol Nanoparticles with Polydopamine-like Coating for PET/SPECT/CT Imaging. ACS Appl Mater Inter.

[131] Lin X, Xie J, Niu G, Zhang F, Gao H, Yang M (2011). Chimeric ferritin nanocages for multiple function loading and multimodal imaging. Nano Lett.

[132] Petersen AL, Binderup T, Jølck RI, Rasmussen P, Henriksen JR, Pfeifer AK (2012). Positron emission tomography evaluation of somatostatin receptor targeted 64Cu-TATE-liposomes in a human neuroendocrine carcinoma mouse model. J Control Release.

[133] Locke LW, Mayo MW, Yoo AD, Williams MB, Berr SS (2012). PET imaging of tumor associated macrophages using mannose coated 64Cu liposomes. Biomaterials.

[134] Lee SB, Lee S-W, Jeong SY, Yoon G, Cho SJ, Kim SK (2017). Engineering of radioiodine-labeled gold core–shell nanoparticles as efficient nuclear medicine imaging agents for trafficking of dendritic cells. ACS Appl Mater Inter.

[135] Gao Z, Hou Y, Zeng J, Chen L, Liu C, Yang W (2017). Tumor microenvironment-triggered aggregation of antiphagocytosis 99mTc‐Labeled Fe3O4 nanoprobes for enhanced tumor imaging in vivo. Adv Mater.

[136] Withers PJ, Bouman C, Carmignato S, Cnudde V, Grimaldi D, Hagen CK (2021). X-ray computed tomography. Nat Reviews Methods Primers.

[137] Viermetz M, Gustschin N, Schmid C, Haeusele J, von Teuffenbach M, Meyer P (2022). Dark-field computed tomography reaches the human scale. Proceed Nat Acad Sci..

[138] Pontico M, Frantellizzi V, Cosma L, De Vincentis G (2020). 111In-Octreoscan SPECT/CT hybrid imaging and 68Ga-DOTANOC PET/CT in neuroendocrine adenoma of the middle ear (NAME). Indian J Radiol Imaging.

[139] Frantellizzi V, Conte M, De Vincentis G, editors. Hybrid imaging of vascular cognitive impairment. Elsevier: Seminars in Nuclear Medicine. 2021. 10.1053/j.semnuclmed.2020.12.006 33353723

[140] Lusic H, Grinstaff MW (2013). X-ray-computed tomography contrast agents. Chem Rev.

[141] de Vries A, Custers E, Lub J, van den Bosch S, Nicolay K, Grüll H (2010). Block-copolymer-stabilized iodinated emulsions for use as CT contrast agents. Biomaterials.

[142] Zheng J, Jaffray D, Allen C (2009). Quantitative CT imaging of the spatial and temporal distribution of liposomes in a rabbit tumor model. Mol Pharm.

[143] Kiessling F, Pichler BJ (2010). Small animal imaging: basics and practical guide.

[144] Dong YC, Hajfathalian M, Maidment PS, Hsu JC, Naha PC, Si-Mohamed S (2019). Effect of gold nanoparticle size on their properties as contrast agents for computed tomography. Sci Rep.

[145] Wang Y, Liu Y, Luehmann H, Xia X, Brown P, Jarreau C (2012). Evaluating the pharmacokinetics and in vivo cancer targeting capability of Au nanocages by positron emission tomography imaging. ACS Nano.

[146] Wang W, Lee NY, Georgi J-C, Narayanan M, Guillem J, Schöder H (2010). Pharmacokinetic analysis of hypoxia 18F-fluoromisonidazole dynamic PET in head and neck cancer. J Nucl Med.

[147] de Barros AB, Tsourkas A, Saboury B, Cardoso VN, Alavi A (2012). Emerging role of radiolabeled nanoparticles as an effective diagnostic technique. EJNMMI Res.

[148] He C, Hu Y, Yin L, Tang C, Yin C (2010). Effects of particle size and surface charge on cellular uptake and biodistribution of polymeric nanoparticles. Biomaterials.

[149] Dreaden EC, Austin LA, Mackey MA, El-Sayed MA (2012). Size matters: gold nanoparticles in targeted cancer drug delivery. Therapeutic delivery.

[150] Faraji AH, Wipf P (2009). Nanoparticles in cellular drug delivery. Bioorg Med Chem.

[151] Zuckerman JE, Choi CHJ, Han H, Davis ME (2012). Polycation-siRNA nanoparticles can disassemble at the kidney glomerular basement membrane. Proceedi Nat Acad  Sci..

[152] Sonavane G, Tomoda K, Makino K (2008). Biodistribution of colloidal gold nanoparticles after intravenous administration: effect of particle size. Colloid Surf B.

[153] Pérez-Campaña C, Gómez-Vallejo V, Puigivila M, Martín A, Calvo-Fernández T, Moya SE (2013). Biodistribution of different sized nanoparticles assessed by positron emission tomography: a general strategy for direct activation of metal oxide particles. ACS Nano.

[154] Gong F, Cheng L, Yang N, Gong Y, Ni Y, Bai S (2020). Preparation of TiH1.924 nanodots by liquid-phase exfoliation for enhanced sonodynamic cancer therapy. Nat Commun.

[155] Tahmasbi Rad A, Chen C-W, Aresh W, Xia Y, Lai P-S, Nieh M-P (2019). Combinational effects of active targeting, shape, and enhanced permeability and retention for cancer theranostic nanocarriers. ACS Appl Mater Inter.

[156] Jain RK, Stylianopoulos T (2010). Delivering nanomedicine to solid tumors. Nat reviews Clin Oncol.

[157] Hobbs SK, Monsky WL, Yuan F, Roberts WG, Griffith L, Torchilin VP (1998). Regulation of transport pathways in tumor vessels: role of tumor type and microenvironment. Proceedi Nat Acad  Sci..

[158] Cabral H, Matsumoto Y, Mizuno K, Chen Q, Murakami M, Kimura M (2011). Accumulation of sub-100 nm polymeric micelles in poorly permeable tumours depends on size. Nat Nanotechnol.

[159] Lv G, Guo W, Zhang W, Zhang T, Li S, Chen S (2016). Near-infrared emission CuInS/ZnS quantum dots: all-in-one theranostic nanomedicines with intrinsic fluorescence/photoacoustic imaging for tumor phototherapy. ACS Nano.

[160] Popović Z, Liu W, Chauhan VP, Lee J, Wong C, Greytak AB (2010). A nanoparticle size series for in vivo fluorescence imaging. Angew Chem.

[161] Guo W, Chen J, Liu L, Eltahan AS, Rosato N, Yu J (2018). Laser-Induced Transformable BiS@ HSA/DTX Multiple Nanorods for Photoacoustic/Computed Tomography Dual-Modal Imaging Guided Photothermal/Chemo Combinatorial Anticancer Therapy. ACS Appl Mater Inter.

[162] Lee JH, Chen KJ, Noh SH, Garcia MA, Wang H, Lin WY (2013). On-demand drug release system for in vivo cancer treatment through self‐assembled magnetic nanoparticles. Angew Chem.

[163] Asanuma D, Sakabe M, Kamiya M, Yamamoto K, Hiratake J, Ogawa M (2015). Sensitive β-galactosidase-targeting fluorescence probe for visualizing small peritoneal metastatic tumours in vivo. Nat Commun.

[164] Song J, Wu B, Zhou Z, Zhu G, Liu Y, Yang Z (2017). Double-layered plasmonic–magnetic vesicles by self‐assembly of Janus amphiphilic gold–iron (II, III) oxide nanoparticles. Angew Chem Int Edit.

[165] Zhao P, Zheng M, Luo Z, Gong P, Gao G, Sheng Z (2015). NIR-driven smart theranostic nanomedicine for on-demand drug release and synergistic antitumour therapy. Sci Rep.

[166] Matsumoto Y, Nichols JW, Toh K, Nomoto T, Cabral H, Miura Y (2016). Vascular bursts enhance permeability of tumour blood vessels and improve nanoparticle delivery. Nat Nanotechnol.

[167] Chen J, Liu L, Motevalli SM, Wu X, Yang XH, Li X (2018). Light-triggered retention and cascaded therapy of albumin‐based theranostic nanomedicines to alleviate tumor adaptive treatment tolerance. Adv Funct Mater.

[168] Champion JA, Mitragotri S (2006). Role of target geometry in phagocytosis. Proceed Nat Acad Sci..

[169] Champion JA, Katare YK, Mitragotri S (2007). Particle shape: a new design parameter for micro-and nanoscale drug delivery carriers. J Control Release.

[170] Champion JA, Mitragotri S (2009). Shape induced inhibition of phagocytosis of polymer particles. Pharmaceut Res.

[171] Doshi N, Mitragotri S (2010). Macrophages recognize size and shape of their targets. PLoS ONE.

[172] Sharma G, Valenta DT, Altman Y, Harvey S, Xie H, Mitragotri S (2010). Polymer particle shape independently influences binding and internalization by macrophages. J Control Release.

[173] Geng Y, Dalhaimer P, Cai S, Tsai R, Tewari M, Minko T (2007). Shape effects of filaments versus spherical particles in flow and drug delivery. Nat Nanotechnol.

[174] Doshi N, Zahr AS, Bhaskar S, Lahann J, Mitragotri S (2009). Red blood cell-mimicking synthetic biomaterial particles. Proceed Nat Acad Sci..

[175] Huang X, Li L, Liu T, Hao N, Liu H, Chen D (2011). The shape effect of mesoporous silica nanoparticles on biodistribution, clearance, and biocompatibility in vivo. ACS Nano.

[176] Muro S, Garnacho C, Champion JA, Leferovich J, Gajewski C, Schuchman EH (2008). Control of endothelial targeting and intracellular delivery of therapeutic enzymes by modulating the size and shape of ICAM-1-targeted carriers. Mol Ther.

[177] Wang G, Inturi S, Serkova NJ, Merkulov S, McCrae K, Russek SE (2014). High-relaxivity superparamagnetic iron oxide nanoworms with decreased immune recognition and long-circulating properties. ACS Nano.

[178] Arnida M, Ray A, Peterson C, Ghandehari H (2011). Geometry and surface characteristics of gold nanoparticles influence their biodistribution and uptake by macrophages. Eur J Pharm Biopharm.

[179] Christian DA, Cai S, Garbuzenko OB, Harada T, Zajac AL, Minko T (2009). Flexible filaments for in vivo imaging and delivery: persistent circulation of filomicelles opens the dosage window for sustained tumor shrinkage. Mol Pharm.

[180] Decuzzi P, Godin B, Tanaka T, Lee S-Y, Chiappini C, Liu X (2010). Size and shape effects in the biodistribution of intravascularly injected particles. J Control Release.

[181] Chauhan VP, Popović Z, Chen O, Cui J, Fukumura D, Bawendi MG (2011). Fluorescent nanorods and nanospheres for real-time in vivo probing of nanoparticle shape‐dependent tumor penetration. Angew Chem.

[182] Black KC, Wang Y, Luehmann HP, Cai X, Xing W, Pang B (2014). Radioactive 198Au-doped nanostructures with different shapes for in vivo analyses of their biodistribution, tumor uptake, and intratumoral distribution. ACS Nano.

[183] Gavze E, Shapiro M (1997). Particles in a shear flow near a solid wall: effect of nonsphericity on forces and velocities. Int J Multiph Flow.

[184] Park J, Butler JE (2010). Analysis of the migration of rigid polymers and nanorods in a rotating viscometric flow. Macromolecules.

[185] Gavze E, Shapiro M (1998). Motion of inertial spheroidal particles in a shear flow near a solid wall with special application to aerosol transport in microgravity. J Fluid Mech.

[186] Gentile F, Chiappini C, Fine D, Bhavane R, Peluccio M, Cheng MM-C (2008). The effect of shape on the margination dynamics of non-neutrally buoyant particles in two-dimensional shear flows. J Biomech.

[187] Lee S-Y, Ferrari M, Decuzzi P (2009). Shaping nano-/micro-particles for enhanced vascular interaction in laminar flows. Nanotechnology.

[188] Liu Z, Cai W, He L, Nakayama N, Chen K, Sun X (2007). In vivo biodistribution and highly efficient tumour targeting of carbon nanotubes in mice. Nat Nanotechnol.

[189] Harris BJ, Dalhaimer P (2012). Particle shape effects in vitro and in vivo. Front Biosci (Schol Ed).

[190] Park JH, von Maltzahn G, Zhang L, Derfus AM, Simberg D, Harris TJ (2009). Systematic surface engineering of magnetic nanoworms for in vivo tumor targeting. small.

[191] Van De Ven AL, Kim P, Fakhoury JR, Adriani G, Schmulen J, Moloney P (2012). Rapid tumoritropic accumulation of systemically injected plateloid particles and their biodistribution. J Control Release.

[192] Godin B, Chiappini C, Srinivasan S, Alexander JF, Yokoi K, Ferrari M (2012). Discoidal porous silicon particles: fabrication and biodistribution in breast cancer bearing mice. Adv Funct Mater.

[193] Chu KS, Hasan W, Rawal S, Walsh MD, Enlow EM, Luft JC (2013). Plasma, tumor and tissue pharmacokinetics of Docetaxel delivered via nanoparticles of different sizes and shapes in mice bearing SKOV-3 human ovarian carcinoma xenograft. Nanomed Nanotechnol Biol Med.

[194] Sun T, Zhang YS, Pang B, Hyun DC, Yang M, Xia Y (2014). Engineered nanoparticles for drug delivery in cancer therapy. Angew Chem Int Edit.

[195] Zhao Z, Ukidve A, Krishnan V, Mitragotri S (2019). Effect of physicochemical and surface properties on in vivo fate of drug nanocarriers. Adv Drug Deliver Rev.

[196] Gessner A, Lieske A, Paulke BR, Müller RH (2003). Functional groups on polystyrene model nanoparticles: influence on protein adsorption. J Biomed Mat Res Part A..

[197] Gessner A, Lieske A, Paulke BR, Müller RH (2002). Influence of surface charge density on protein adsorption on polymeric nanoparticles: analysis by two-dimensional electrophoresis. Eur J Pharm Biopharm.

[198] Semple SC, Chonn A, Cullis PR (1998). Interactions of liposomes and lipid-based carrier systems with blood proteins: Relation to clearance behaviour in vivo. Adv Drug Deliver Rev.

[199] Duan X, Li Y (2013). Physicochemical characteristics of nanoparticles affect circulation, biodistribution, cellular internalization, and trafficking. Small.

[200] Xiao K, Li Y, Luo J, Lee JS, Xiao W, Gonik AM (2011). The effect of surface charge on in vivo biodistribution of PEG-oligocholic acid based micellar nanoparticles. Biomaterials.

[201] Cho EC, Xie J, Wurm PA, Xia Y (2009). Understanding the role of surface charges in cellular adsorption versus internalization by selectively removing gold nanoparticles on the cell surface with a I2/KI etchant. Nano Lett.

[202] Arvizo RR, Miranda OR, Thompson MA, Pabelick CM, Bhattacharya R, Robertson JD (2010). Effect of nanoparticle surface charge at the plasma membrane and beyond. Nano Lett.

[203] Fröhlich E (2012). The role of surface charge in cellular uptake and cytotoxicity of medical nanoparticles. Int J Nanomed.

[204] Kim ST, Saha K, Kim C, Rotello VM (2013). The role of surface functionality in determining nanoparticle cytotoxicity. Acc Chem Res.

[205] Campbell F, Bos FL, Sieber S, Arias-Alpizar G, Koch BE, Huwyler (2018). Directing nanoparticle biodistribution through evasion and exploitation of Stab2-dependent nanoparticle uptake. ACS Nano.

[206] Hung C-C, Huang W-C, Lin Y-W, Yu T-W, Chen H-H, Lin S-C (2016). Active tumor permeation and uptake of surface charge-switchable theranostic nanoparticles for imaging-guided photothermal/chemo combinatorial therapy. Theranostics.

[207] Yuan YY, Mao CQ, Du XJ, Du JZ, Wang F, Wang J (2012). Surface charge switchable nanoparticles based on zwitterionic polymer for enhanced drug delivery to tumor. Adv Mater.

[208] Wang H-X, Zuo Z-Q, Du J-Z, Wang Y-C, Sun R, Cao Z-T (2016). Surface charge critically affects tumor penetration and therapeutic efficacy of cancer nanomedicines. Nano Today.

[209] Thurston G, McLean JW, Rizen M, Baluk P, Haskell A, Murphy TJ (1998). Cationic liposomes target angiogenic endothelial cells in tumors and chronic inflammation in mice. J Clin Investig.

[210] Song W, Popp L, Yang J, Kumar A, Gangoli VS, Segatori L (2015). The autophagic response to polystyrene nanoparticles is mediated by transcription factor EB and depends on surface charge. J Nanobiotechnol.

[211] Arias-Alpizar G, Kong L, Vlieg RC, Rabe A, Papadopoulou P, Meijer MS (2020). Light-triggered switching of liposome surface charge directs delivery of membrane impermeable payloads in vivo. Nat Commun.

[212] Harris JM, Martin NE, Modi M, Pegylation (2001). Clin Pharmacokinet.

[213] Adams ML, Lavasanifar A, Kwon GS (2003). Amphiphilic block copolymers for drug delivery. J Pharm Sci.

[214] He X, Nie H, Wang K, Tan W, Wu X, Zhang P (2008). In vivo study of biodistribution and urinary excretion of surface-modified silica nanoparticles. Anal Chem.

[215] Daou TJ, Li L, Reiss P, Josserand V, Texier I (2009). Effect of poly (ethylene glycol) length on the in vivo behavior of coated quantum dots. Langmuir.

[216] Li C, Li F, Zhang Y, Zhang W, Zhang X-E, Wang Q (2015). Real-time monitoring surface chemistry-dependent in vivo behaviors of protein nanocages via encapsulating an NIR-II Ag2S quantum dot. ACS Nano.

[217] Khargharia S, Kizzire K, Ericson MD, Baumhover NJ, Rice KG (2013). PEG length and chemical linkage controls polyacridine peptide DNA polyplex pharmacokinetics, biodistribution, metabolic stability and in vivo gene expression. J Control Release.

[218] Li S, Chen H, Liu H, Liu L, Yuan Y, Mao C (2020). In Vivo Real-Time Pharmaceutical Evaluations of Near-Infrared II Fluorescent Nanomedicine Bound Polyethylene Glycol Ligands for Tumor Photothermal Ablation. ACS Nano.

[219] Jokerst JV, Lobovkina T, Zare RN, Gambhir SS (2011). Nanoparticle PEGylation for imaging and therapy. Nanomedicine.

[220] Mosqueira VCF, Legrand P, Morgat J-L, Vert M, Mysiakine E, Gref R (2001). Biodistribution of long-circulating PEG-grafted nanocapsules in mice: effects of PEG chain length and density. Pharmaceut Res.

[221] Perry JL, Reuter KG, Kai MP, Herlihy KP, Jones SW, Luft JC (2012). PEGylated PRINT nanoparticles: the impact of PEG density on protein binding, macrophage association, biodistribution, and pharmacokinetics. Nano Lett.

[222] Hak S, Helgesen E, Hektoen HH, Huuse EM, Jarzyna PA, Mulder WJ (2012). The effect of nanoparticle polyethylene glycol surface density on ligand-directed tumor targeting studied in vivo by dual modality imaging. ACS Nano.

[223] Liu X, Tao H, Yang K, Zhang S, Lee S-T, Liu Z (2011). Optimization of surface chemistry on single-walled carbon nanotubes for in vivo photothermal ablation of tumors. Biomaterials.

[224] Gbadamosi J, Hunter A, Moghimi SM (2002). PEGylation of microspheres generates a heterogeneous population of particles with differential surface characteristics and biological performance. FEBS Lett.

[225] Avgoustakis K, Beletsi A, Panagi Z, Klepetsanis P, Livaniou E, Evangelatos G (2003). Effect of copolymer composition on the physicochemical characteristics, in vitro stability, and biodistribution of PLGA–mPEG nanoparticles. Int J Pharm.

[226] Mura S, Nicolas J, Couvreur P (2013). Stimuli-responsive nanocarriers for drug delivery. Nat Mater.

[227] Liu J, Bu J, Bu W, Zhang S, Pan L, Fan W (2014). Real-time in vivo quantitative monitoring of drug release by dual‐mode magnetic resonance and upconverted luminescence imaging. Angew Chem.

[228] Mathijssen RH, Sparreboom A, Verweij J (2014). Determining the optimal dose in the development of anticancer agents. Nat reviews Clin Oncol.

[229] Greco F, Vicent MJ (2009). Combination therapy: opportunities and challenges for polymer–drug conjugates as anticancer nanomedicines. Adv Drug Deliver Rev.

[230] Chen C-Y, Kim TH, Wu W-C, Huang C-M, Wei H, Mount CW (2013). pH-dependent, thermosensitive polymeric nanocarriers for drug delivery to solid tumors. Biomaterials.

[231] Wood CA, Han S, Kim CS, Wen Y, Sampaio DRT, Harris JT (2021). Clinically translatable quantitative molecular photoacoustic imaging with liposome-encapsulated ICG J-aggregates. Nat Commun.

[232] Zhang Y, Yin Q, Yen J, Li J, Ying H, Wang H (2015). Non-invasive, real-time reporting drug release in vitro and in vivo. Chem Commun.

[233] Li X, Bottini M, Zhang L, Zhang S, Chen J, Zhang T (2018). Core–satellite nanomedicines for in vivo real-time monitoring of enzyme-activatable drug release by fluorescence and photoacoustic dual-modal imaging. ACS Nano.

[234] Yan C, Guo Z, Liu Y, Shi P, Tian H, Zhu W-H (2018). A sequence-activated AND logic dual-channel fluorescent probe for tracking programmable drug release. Chem Sci.

[235] Zhu X, Li J, Peng P, Hosseini Nassab N, Smith BR (2019). Quantitative drug release monitoring in tumors of living subjects by magnetic particle imaging nanocomposite. Nano Lett.

[236] Zhang Z, Wells CJ, King AM, Bear JC, Davies G-L, Williams GR (2020). pH-Responsive nanocomposite fibres allowing MRI monitoring of drug release. J Mater Chem B.

[237] Wang S, Zhou Z, Wang Z, Liu Y, Jacobson O, Shen Z (2019). Gadolinium metallofullerene-based activatable contrast agent for tumor signal amplification and monitoring of drug release. Small.

